# Electrical Stimulation of Human Adipose Tissue-Derived Mesenchymal Stem Cells and Schwann Cells for Regulating Extracellular Vesicle Biogenesis and Inflammation

**DOI:** 10.3390/bioengineering13091025

**Published:** 2026-09-02

**Authors:** Danyale Berry, Aakash Nathani, Fernando Carrillo, Abby Scott, Justice Ene, Colin Esmonde, Mandip Singh, Yan Li, Changchun Zeng

**Affiliations:** 1 Department of Industrial and Manufacturing Engineering, FAMU-FSU College of Engineering, Florida Agricultural and Mechanical University, Tallahassee, FL 32310, USA; danyale1.berry@famu.edu; 2 High Performance Materials Institute, FAMU-FSU College of Engineering, Florida State University, Tallahassee, FL 32310, USA; 3 College of Pharmacy and Pharmaceutical Sciences, Florida Agricultural and Mechanical University, Tallahassee, FL 32307, USA; aakash1.nathani@famu.edu (A.N.); mandip.sachdeva@famu.edu (M.S.); 4 Department of Chemical and Biomedical Engineering, FAMU-FSU College of Engineering, Florida State University, 2525 Pottsdamer St., Tallahassee, FL 32310, USA; fc20a@fsu.edu (F.C.); ars20@fsu.edu (A.S.); je17d@fsu.edu (J.E.); cme18b@fsu.edu (C.E.)

**Keywords:** human adipose-derived mesenchymal stem cells, human Schwann cells, electrical stimulation, extracellular vesicles, EV biogenesis, peripheral neuropathy

## Abstract

Peripheral neuropathy (PN) is a debilitating condition characterized by chronic pain, numbness, and motor dysfunction, with limited treatment options. Ischemic stroke can cause central neuropathy, which may also induce PN. Human mesenchymal stem cells (hMSCs) have shown promise in therapeutic applications, but limitations in cell viability, immune response, and efficacy persist. Extracellular vesicles (EVs), which facilitate cell-free intercellular communication, offer a promising alternative for nerve regeneration. Electrical stimulation (ES) has emerged as a method to enhance EV secretion, and this study investigates its potential for promoting EV production from human adipose tissue-derived mesenchymal stem cells (hASCs) and human Schwann cells (hSCs). In this study, hASCs, hSCs, and lipopolysaccharide (LPS)-induced inflamed hSCs were subjected to one hour of low-frequency direct current (DC) electrical stimulation (100 mV/mL) for 7 days. EVs were isolated using differential ultracentrifugation and characterized through nanoparticle tracking analysis (NTA). Gene expression was analyzed via qRT-PCR to evaluate markers associated with EV biogenesis as well as pro- and anti-inflammatory cytokines. Our results demonstrate that ES significantly increases EV secretion from both hASCs and hSCs, with a notable upregulation of genes involved in both the endosomal sorting complex required for transport (ESCRT)-dependent and ESCRT-independent pathways of EV biogenesis. Additionally, ES modulates inflammation-related markers, promoting anti-inflammatory gene expression and reducing pro-inflammatory gene levels. Notably, LPS-induced hSCs exhibited a phenotype shift from myelinating to non-myelinating cells, producing EVs capable of modulating the inflammatory microenvironment. However, prolonged exposure to ES led to a decrease in EV secretion and changes in EV size distribution, suggesting potential cellular adaptation or membrane stress. This study highlights the potential of ES as a scalable, cell-free strategy to enhance EV production, offering new insights into its therapeutic applications for peripheral neuropathy and nerve regeneration.

## 1. Introduction

The peripheral nervous system (PNS) is highly susceptible to trauma, leading to chronic pain, numbness, and motor dysfunction associated with peripheral neuropathy. Current symptom-focused therapies often fail to prevent further nerve damage. An ischemic stroke can cause central neuropathy, known as central post-stroke pain (CPSP), which may also induce PN. Human mesenchymal stem cells (hMSCs) have emerged as a promising alternative due to their regenerative and immunomodulatory capabilities [1]. hMSCs secrete trophic factors and cytokines that downregulate pro-inflammatory gene expression, modulate the local microenvironment, and support neuronal survival and axonal regrowth [2]. Preclinical studies demonstrate that hMSC transplantation improves motor recovery and reduces neuroinflammation, highlighting their ability to restore metabolically active, viable cells following injury [3].

Human Schwann cells (hSCs), the principal glial cells of the PNS, play a pivotal role in neuronal repair. Following injury, hSCs exhibit remarkable plasticity, dedifferentiating into a repair phenotype (rSCs) that clears axonal debris, guides regenerating axons via Büngner bands, and coordinates macrophage polarization from an M1 to an M2 phenotype [3,4,5]. Additionally, rSCs secrete growth factors such as nerve growth factor (NGF) and brain-derived neurotrophic factor (BDNF) to support axonal regrowth and remyelination [6]. Recent transplantation studies show that hSCs restore electrophysiological and behavioral function in animal models, establishing them as essential cellular targets for peripheral nerve repair [7].

Beyond direct cell transplantation, extracellular vesicles (EVs) derived from hMSCs and hSCs offer a compelling cell-free therapeutic strategy. While cell-based therapies face challenges regarding long-term viability, immune rejection, and manufacturing scalability, EVs have emerged as a scalable, cell-free therapeutic strategy [8,9,10,11]. EVs (~30–200 nm) encapsulate biologically active cargo—including miRNAs, mRNAs, lipids, proteins, and cytokines—that drive intracellular communication and tissue homeostasis [11,12,13,14,15,16]. Importantly, hMSC- and hSC-derived EVs replicate key parental therapeutic effects, including suppression of inflammation, enhancement of angiogenesis, and promotion of neuronal recovery in neuroinflammatory and ischemic stroke models [11,12,13].

Preclinical studies demonstrate the potent therapeutic impact of hMSC-derived EVs across disease models. For instance, EV administration reduces demyelination and neuroinflammation in models of multiple sclerosis (MS), leading to functional recovery [17]. Similarly, miRNA-enriched EVs have been shown to target injured nerves, suppress Toll-like receptor (TLR)-4/NF-kB inflammatory signaling and improve nerve conduction velocity and sensory responses in diabetic peripheral neuropathy models [11]. These findings underscore the therapeutic promise of EVs as a safer, cell-free alternative to live-cell transplantation.

A primary bottleneck in translating EV therapies lies in achieving sufficient yield and potency for clinical applications. While mechanical stimulation via bioreactors enables high-volume production, high shear stress and energy dissipation rates can subject cells to physical strain, leading to fragmentation, phenotypic shifts, and heterogenous EV populations [15,18]. Similarly, metabolic modulation via hypoxia is widely used to boost EV yield, but its reproducibility remains debated [19]. Hypoxia-based protocols suffer from extreme sensitivity to oxygen concentration gradients governed by atmospheric pressure, cellular metabolic demand, and media depth [19]. This lack of standardization makes consistent, large-scale EV production challenging.

Electrical stimulation (ES) provides a direct, non-invasive method to modulate EV biogenesis without traditional environmental stressors. ES modulates the cellular membrane potential, thus inducing intracellular calcium influx, which activates key master regulators of vesicle trafficking and exocytosis (e.g., PI3K/Akt, MAPK/ERK, NF-kB) [20]. During electrotaxis, differential polarization—cathode-facing depolarization and anode-facing hyperpolarization—prompts localized ion influx and cytoskeletal reorganization that actively drives vesicle budding and release [20,21,22,23,24,25]. However, the efficacy of ES depends heavily on parameter optimization. Overstimulation with high current or prolonged direct current (DC) exposure generates excess heat, depletes nutrients, and damages plasma membranes, leading to reduced cell viability [24]. In contrast, low-frequency ES safely alters membrane potential, enhances EV release, upregulates neural markers, and promotes neurite outgrowth [26,27,28,29]. Despite these advantages, a fundamental knowledge gap remains regarding how standardized ES protocols affect EV biogenesis kinetics and immunomodulatory cargo across distinct peripheral nerve cell populations.

This study investigates whether low-frequency DC ES can serve as a versatile platform to enhance EV production across key cellular components for nerve regeneration. We evaluated the stimulatory effects of low-frequency DC ES on human adipose-derived MSCs (hASCs) and hSCs. This study assessed hASCs as a primary “bio-factory” for therapeutic EVs, while utilizing hSCs—under both homeostatic and LPS-induced inflammatory conditions—as a functional proof of concept for ES parameter versatility in a stimulated injury environment. It was hypothesized that low-frequency ES would initially surge EV secretion during early stimulation and then plateau as cells adapted, while still maintaining parent cell viability and downstream bioactivity. To provide a comprehensive “Phenotype-to-Mechanism” analysis, the resulting EV yields and size distributions, biogenesis machinery, and immunomodulatory profiles were assessed. This work advances ES as a scalable, cell-type-independent strategy for peripheral nerve regeneration.

## 2. Materials and Methods

### 2.1. Cell Culture

#### 2.1.1. HASC Cultures

hASCs (RoosterBio Inc., Frederick, MD, USA; SKU: C46001AD, Donor: 310330) were cryopreserved in Alpha Minimum Essential Medium (αMEM, Thermo Scientific, Waltham, MA, USA; Cat. No. 11900073) supplemented with 10% fetal bovine serum (FBS, Life Technologies, Carlsbad, CA, USA) and 5% dimethyl sulfoxide (DMSO, Sigma-Aldrich, St. Louis, MO, USA). Cells were thawed at passage 3 and expanded to passage 5 to ensure a robust and homogeneous population for all downstream experiments. Cultures were maintained in complete culture media (CCM) composed of αMEM, 10% FBS, and 1% penicillin/streptomycin (Life Technologies) under standard incubator conditions (37 °C, 5% CO_2_, 20% O_2_) with media exchanges performed every 3 days. Upon reaching 80% confluency, hASCs were seeded at a density of 50,000 cells/well in 6-well plates. To facilitate direct comparison with subsequent hSC experiments, a standardized stimulation and collection timeline was adopted. On day 0, cells were acclimated in CCM. After 24 h (day 1), the media were replaced with EV-depleted CCM. Conditioned media were collected for EV isolation on days 2, 5, and 7.

#### 2.1.2. hSC Culture and LPS-Induced Inflammatory Conditions

**Figure 1:**
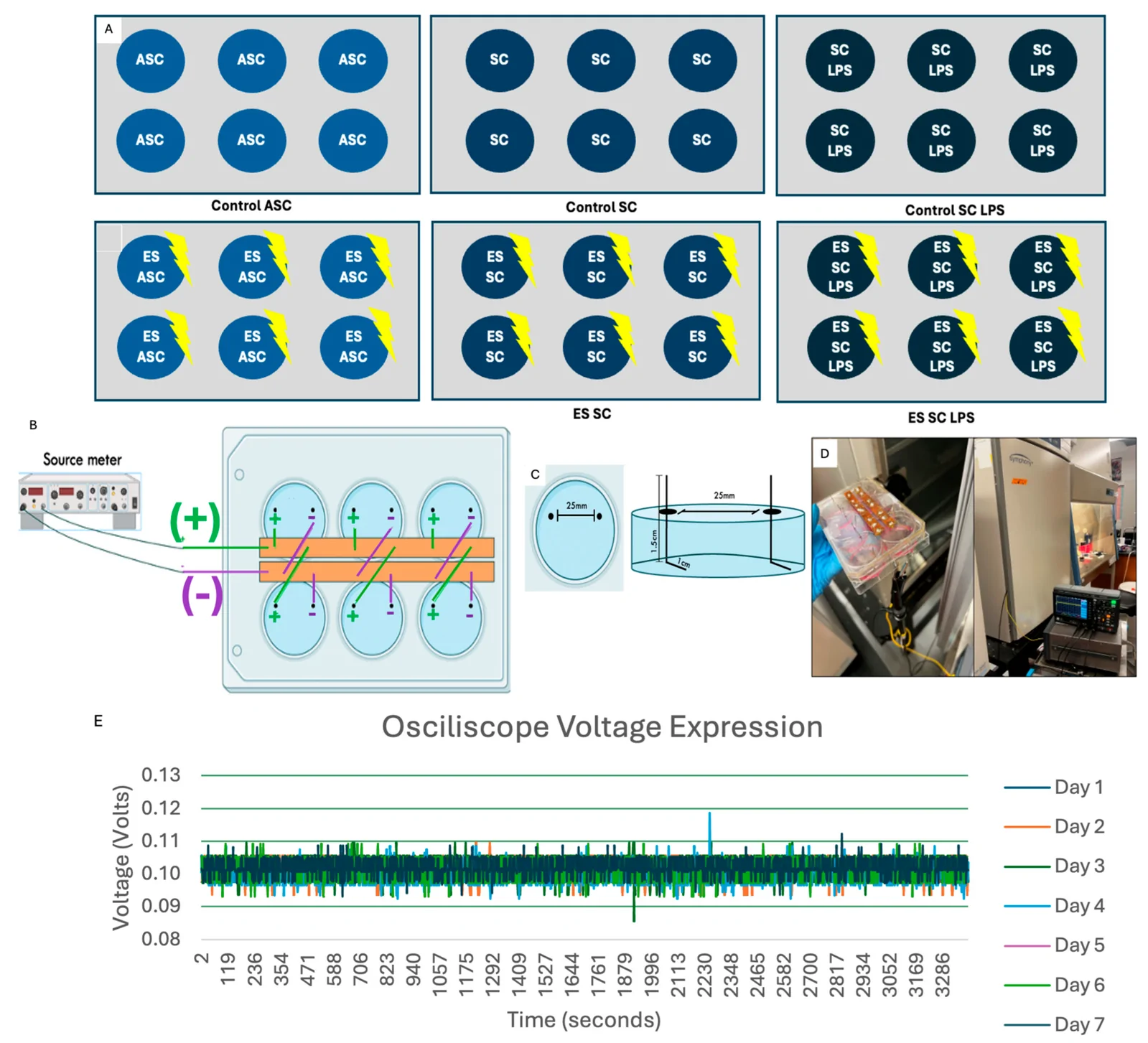
Design and validation of the ES system for analysis. (**A**) Experimental design comparing control (cells without ES) and electrically stimulated hASCs and hSCs under basal and inflammatory (LPS) conditions. (**B**) Copper wire configuration of the ES chamber connected to the source meter, showing the setup for simultaneous multi-well stimulation. (**C**) Electrode geometry showing platinum electrodes positioned at a fixed 25 mm distance within each well to ensure a uniform and reproducible electrical field. (**D**) Operational setup of the ES chamber during incubation, confirming the system’s compatibility with standard cell culture environments. (**E**) Voltage characterization demonstrating that the system maintains long-term stability with minimal drift over the 7-day experimental window.

hSCs (ATCC, Manassas, VA, USA; Cat. No. CRL-3392), as described in our previous study [30], were cryopreserved in high-glucose Dulbecco’s Modified Eagle Medium (DMEM, Life Technologies), 10% FBS, and 5% DMSO. Cells were thawed at passage 8 and expanded in growth media consisting of high-glucose DMEM, 10% FBS, and 1% penicillin/streptomycin. Once cultures reached 80% confluency, hSCs were seeded into 6-well plates at 50,000 cells/well. To evaluate the effects of electrical and inflammatory stimuli, four experimental groups were established: (1) control (no ES, no LPS), (2) ES-only, (3) LPS only (500 ng/mL), and (4) combined treatment (ES + LPS) (Figure 1A). Following the same standardized timeline as the hASC groups, media were replaced on day 1 with EV-depleted high-glucose DMEM (Thermo Scientific; Cat. No. 11965092). For inflammatory groups, LPS (Sigma-Aldrich) was replenished with each media change on days 2, 5, and 7 to maintain a consistent inflammatory environment. Conditioned media and cell lysates were harvested on day 7 for comprehensive downstream analysis.

### 2.2. Electrical Stimulation Chamber Construction

#### 2.2.1. Chamber Design and Fabrication

The ES chamber was custom-fabricated using a standard polystyrene 6-well plate lid, modified to deliver uniform stimulation across all wells connected in series. The design, inspired by Leppik et al. [31], incorporates 99.99% pure platinum wire electrodes (0.368 mm diameter, Thermo Scientific; Cat. No. 013039.BU), high-purity copper foil strips (0.1 mm thickness; commercial supplier), and insulated copper wiring (Figure 1B). High-purity platinum was specifically selected due to its electrochemical inertness, high charge-injection capacity, and resistance to corrosion, effectively preventing the release of toxic metal ions or electrochemical byproducts into the media under low DC potentials.

To accommodate the electrodes, two 0.015-inch diameter holes were precisely drilled at fixed inter-electrode distance of 25 mm (2.5 cm) within each well (12 holes total). Platinum wires were cut into 2.5 cm segments and bent into an L-shape at the 1 cm mark to ensure full immersion and stable, parallel alignment above the cell culture monolayer (Figure 1C).

For consistent current delivery, copper foil strips were applied along the lid to link all wells in series—one strip connecting the positive (anode) electrodes and the other connecting the negative (cathode) electrodes. Connection points were sanded with fine-grit sandpaper and soldered to ensure optimal conductivity. To validate stimulation uniformly across the assembly, circuit connectivity was verified using a digital multimeter and oscilloscope, confirming an equal, consistent voltage drop across each individual well connected in series (Figure 1D,E).

#### 2.2.2. Sterilization and Reusability Procedures

To ensure sterility and maintain functionality across multiple uses, the ES chamber was subjected to a standardized sterilization procedure prior to each stimulation session. First, the entire chamber lid and embedded electrodes were submerged in 70% isopropanol alcohol (IPA) for 30 min. Following IPA sterilization, the components were rinsed in sterile phosphate-buffered saline (PBS) to remove any residual alcohol, which could be cytotoxic to the cell culture. After the PBS rinse, the chamber and electrodes were further rinsed with sterile deionized (DI) water to remove any remaining salts from the PBS. The components were then dried under UV light in a biosafety cabinet for 30 min to ensure complete drying and sterilization.

Between experimental runs, the platinum electrodes were first washed in a sterile 6-well plate filled with 70% IPA to eliminate residual biological material. The electrodes were then transferred to a separate 6-well plate containing sterile PBS to remove any remaining IPA. After this, the electrodes were rinsed with sterile DI water to ensure all PBS and IPA residue were removed. Finally, the electrodes and lid were dried under UV light within the biosafety cabinet for 30 min before being stored overnight. The electrodes were stored in a closed well plate within the biosafety cabinet to maintain sterility until the next use. This protocol allowed for repeated use of the ES chamber without compromising sterility or electrical performance.

### 2.3. Electrical Stimulation Experimental Design and Methods

#### 2.3.1. Study Design and Rational

The objective of this study is to evaluate the feasibility of ES as a method to enhance EV biogenesis in cell types central to peripheral nerve regeneration. To evaluate production yield and therapeutic potential, hASCs and hSCs were tested under basal and challenged conditions. By utilizing hSCs treated with LPS, the study models the acute inflammatory microenvironment conditions observed in patients with PN. This enables an evaluation of the immunomodulatory shifts in EV secretion and gene expression under stress. While initial assessments focused on hASC EV yield, the scope expanded to include a comprehensive comparison of stimulated and unstimulated populations across all three groups (hASC, hSC, and hSC exposed to LPS). The 7-day study duration with media collections on days 2, 5, and 7 was established during preliminary optimization to synchronize feeding schedules between cell lines, ensure adequate nutrient supply for metabolically demanding hSCs, and prevent culture hyper-confluency, which would introduce stress-related transcriptional artifacts.

#### 2.3.2. Cell Seeding and Culture Maintenance

On day 0, passage five hASCs and passage 8 hSCs were seeded at a density of 50,000 cells per well into 6-well plates. hASCs were maintained in αMEM supplemented with 10% FBS and 5% penicillin/streptomycin (CCM), while hSCs utilized high-glucose DMEM with identical supplementation. Following a 24 h acclimation period, culture media were replaced with EV-depleted media on day 1. For the inflammatory model groups, hSCs were treated with 500 ng/mL of LPS (Sigma), a concentration previously shown to effectively induce a pro-inflammatory phenotype in Schwann cells without significantly compromising cell viability [32]. The LPS was reintroduced with each media change to maintain a consistent inflammatory stimulus throughout the 7-day study duration.

#### 2.3.3. Experimental Procedure

Experimental groups were subjected to daily ES for one hour over a 7-day period using a Keysight DC Power Supply (E63613A, Triple Output). The power supply delivered a constant direct current potential of 100 mV per well. Based on the 25 mm (2.5 cm) fixed electrode distance established in Section 2.2.1, this setup generated a uniform, low-intensity electric field strength of 40 mV/cm (E = V/d) across the cell growth plane. To safeguard against resistive heating, the current compliance on the power supply was capped at a maximum limit of 2 mA. Output voltage stability was monitored during stimulation using the oscilloscope connected directly to the external terminal leads.

Prior to each session, standard culture lids were replaced with the sterilized ES lids containing platinum electrodes inside the biosafety cabinet. The assembly was connected to the power supply and an oscilloscope and returned to the incubator for the pre-set one-hour stimulation period. Following stimulation, ES lids were exchanged for standard lids under sterile conditions, and the cells were returned to the incubator to recover overnight. The electrodes were sanitized and prepared for the next stimulation session. To maintain metabolic stability and accommodate the nutrient requirements of the cell populations, culture media were refreshed and collected for EV isolation on days 2, 5, and 7. Cell morphology and general culture health were documented before each stimulation session. After 7 days, the cells were harvested for downstream transcriptomic and functional analyses.

#### 2.3.4. Sample Collection

At the conclusion of the 7-day electrical stimulation period, the conditioned media from each group were collected and stored at −80 °C for subsequent EV isolation. Following media collection, adherent cells were gently washed twice with sterile PBS to remove residual proteins. Trypsin was then added and incubated at 37 °C for 5 min to detach adherent cells, followed by neutralization with an equal volume of CCM. The resulting cell suspension was centrifuged at 500 *g* for 5 min to pellet the cells. The supernatant was discarded, and the cell pellet was stored at −80 °C for RNA extraction and qRT-PCR. qRT-PCR was utilized to analyze the expression of EV biogenesis markers, as well as pro- and anti-inflammatory gene expression across all experimental conditions.

### 2.4. EV Isolation and Characterization

#### 2.4.1. EV Isolation Through Differential Ultracentrifugation

EVs derived from each culture condition were isolated using differential ultracentrifugation. Briefly, collected conditioned media were first centrifuged at 500 *g* for 5 min at 4 °C to remove cellular debris. The supernatant was collected and subjected to a second centrifugation at 2000 *g* for 10 min at 4 °C, followed by a third spin at 10,000 *g* for 30 min at 4 °C to eliminate larger vesicles and remaining debris. The resulting supernatant was mixed by inversion with a 16% (*w*/*v*) polyethylene glycol (PEG) solution at a 1:1 volume ratio and incubated overnight at 4 °C to precipitate EVs. The next day, the solution was centrifuged at 10,000 *g* for 70 min at 4 °C. Supernatants were discarded, and each pellet was then resuspended in 1 mL of particle-free PBS and ultracentrifuged at 100,000 *g* for 2 h at 4 °C. The final supernatant was carefully removed, and each pellet was resuspended in 200 μL of PBS. Isolated EVs were stored at −80 °C until further characterization.

#### 2.4.2. Nanoparticle Tracking Analysis (NTA)

To determine EV size distribution and particle concentration, NTA was performed on samples isolated by differential ultracentrifugation using the ZetaView^®^ (TWIN PMX-220, Ammersee, Germany) instrument. For each measurement, 20 μL of the EV sample was diluted in 10 mL of particle-free PBS, resulting in a 1:500 dilution. ZetaView^®^ software (version 8.05.11 SP4) was used to calculate the mean and mode particle size, as well as the concentration per mL of solution. Measurements were performed in triplicate to ensure accuracy, and the chamber was thoroughly rinsed with particle-free PBS between samples to prevent cross-contamination. The NanoSight LM10-HS instrument (Malvern Instruments, Malvern, UK) was also used. It was configured with a blue laser (488 nm) and an sCMOS camera. The samples were diluted 1:1000 in filtered PBS. Three videos of 60 s were captured with the camera shutter speed fixed at 30.00 ms. The camera level was set to 12, and the detection threshold was set to 5. The collected videos were analyzed using NTA3.4 software to obtain the mode and mean size distributions, as well as the concentration of particles. Compared to the mean size, the mode size is usually a more accurate representation because the vesicle aggregates may affect the value of the mean size.

#### 2.4.3. Western Blot Analysis

Western blotting was performed to verify the presence of EVs by detecting characteristic EV-associated protein markers. Total protein concentration in EV samples was quantified using the Bradford assay. Briefly, EVs were lysed in radio-immunoprecipitation assay (RIPA) buffer supplemented with bovine serum albumin and incubated with the Bradford reagent in a 96-well plate at room temperature for 5–10 min. Absorbance was measured at 660 nm using a microplate reader, and protein concentration were standardized to 20 µg/mL across all samples. Proteins were denatured at 95 °C for 10 min, separated via SDS-PAGE at 150 V for 2 h, and transferred to a membrane. Transfer efficiency was confirmed with Ponceau staining. Membranes were then blocked for 1 h in 5% skim milk (*w*/*v*) prepared in Tris-buffered saline with 0.1% Tween 20 (TBST), followed by overnight incubation at 4 °C with primary antibodies targeting EV markers (Supplementary Table S1). The following day, membranes were washed with TBST, incubated with appropriate secondary antibodies for one hour, and washed again. Protein bands were visualized using chemiluminescent detection on the Li-COR Odyssey imaging system after membranes were sealed in plastic lamination.

#### 2.4.4. Transmission Electron Microscopy (TEM)

TEM was utilized to visualize and confirm the morphology, size, and structural integrity of the isolated EVs, following the protocol described previously in our group [33]. This high-resolution imaging technique enables visualization of lipid bilayer-enclosed vesicles. EV pellets were resuspended in 50–100 µL of sterile-filtered PBS to preserve particle stability. For sample preparation, 5 µL of the EV suspension was pipetted onto parafilm to minimize sample loss. Carbon-coated 400 hex mesh copper grids (Electron Microscopy Sciences, EMS, Hatfield, PA, USA) were placed, coating side down, onto the droplets using fine-tip forceps and incubated at room temperature for 1 h to allow particle absorption. Following absorption, the grids were gently washed three times with sterile-filtered PBS to remove unbound material. Samples were then fixed in 2% paraformaldehyde (EM grade) for 10 min at room temperature to preserve vesicle structure. Grids were subsequently transferred onto a 20 µL drop of 2.5% glutaraldehyde (EM grade) and incubated for an additional 10 min to increase image contrast. To further stabilize and embed the EVs, grids were incubated in a solution of 0.13% methyl cellulose and 0.4% uranyl acetate for 10 min. Excess solution was carefully removed, and the grids were left to air dry. Images were acquired using a Hitachi HT7800 transmission electron microscope at Florida State University [34], confirming the presence of spherical EVs with distinct lipid bilayer membranes.

### 2.5. Quantitative Reverse Transcription Polymerase Chain Reaction (qRT-PCR) Analysis

Following ES experiments, harvested cell pellets from both hASC and hSC treatment groups were stored at −80 °C prior to RNA extraction to preserve transcript integrity. Total mRNA was extracted using the RNeasy Plus Kit (Qiagen, Valencia, CA, USA) following the manufacturer’s protocol, which includes on-column DNA removal to ensure high-purity, DNA-free RNA. RNA quality and concentration were assessed using a Nanodrop spectrophotometer (Thermo Scientific), and only samples with A260/A280 ratios between 1.8 and 2.2 were used for downstream analysis. Complementary DNA synthesis was performed using 2 ng of total RNA, anchored oligo-dT primers (Operon, Huntsville, AL, USA), and SuperScript III (Invitrogen, Carlsbad, CA, USA), following the manufacturer’s guidelines. Primers specific to genes of interest were designed using Oligo Explorer 1.2 (GeneLink, Hawthorne, NY, USA). Gene expression was quantified using SYBR Green PCR Master Mix (Applied Biosystems, Foster City, CA, USA) on an AB17500 real-time PCR system. The qPCR thermal cycling conditions were: 2 min at 50 °C, 10 min at 95 °C, followed by 40 cycles of 95 °C for 15 s, 55 °C for 30 s, and 68 °C for 30 s, ending with a melt curve analysis. Target genes included those involved in EV biogenesis through the endosomal sorting complex required for transport (ESCRT)-dependent and ESCRT-independent pathways, as well as pro- and anti-inflammatory markers (Supplementary Table S2), to assess the regulation of EV secretion and the cells’ immunomodulatory profile under different conditions. For all samples, β-actin (ACTB) was used as the endogenous control. The comparative Ct (ΔΔCt) method was employed to calculate relative gene expression. In hASC samples, gene expressions were normalized to unstimulated ES hASCs. hSC samples were normalized to their respective hSC unstimulated controls.

### 2.6. EV Treatment in an In Vitro Ischemic Stroke Model

#### 2.6.1. An In Vitro Ischemic Stroke Model Treated by EVs

To establish the therapeutic baseline of hASC-derived EVs within a neurologically relevant context, an in vitro ischemic stroke model was utilized through oxygen and glucose deprivation (OGD). EVs were incorporated into collagen hydrogels at defined concentrations (1 × 10^9^, 5 × 10^9^, or 1 × 10^10^ EVs/mL) to evaluate sustained-release kinetics and neuroprotective outcomes. hASCs seeded onto the hydrogel surface were subjected to hypoxic stress for 6, 12, or 24 h. Following exposure, cultures were transferred to EV-depleted media overnight to ensure that metabolic recovery was solely attributed to the hydrogel-released dosages. For each hypoxia duration, 36 wells in a 96-well plate were analyzed, comprising 9 wells per treatment group. This arrangement enabled triplicate measurements for three outcome assays: the 3-(4,5-dimethylthiazol-2-yl)-2,5-diphenyltetrazolium bromide (MTT) assay, to evaluate mitochondrial activity and cell viability; the lactate dehydrogenase (LDH) assay, to assess membrane integrity and cytotoxicity; and the reactive oxygen species (ROS) assay, to quantify oxidative stress, relative to the control group. This multi-assay approach provides a comprehensive profile of hASC-EV efficacy. Once data collection was complete, statistical analyses were performed using two-tailed, type II t-tests to determine significance for each of the two independent variables: EV concentration and hypoxic exposure time.

#### 2.6.2. MTT Assay

MTT stock solution (5 mg/mL, Sigma-Aldrich; Cat. No. M2128) was diluted in cell culture media at 1:10 and was added to 96-well plates containing hydrogel-encapsulated hASCs subjected to hypoxia and EV treatment, and incubated at 37 °C for 4 h. Following incubation, the formazan crystals were gently washed with PBS and solubilized in DMSO. Samples were transferred into microcentrifuge tubes and centrifuged at 800 *g* for 5 min. A 50 μL aliquot of the supernatant was transferred to a clean 96-well plate, and absorbance was measured by a Cytation 5 Cell Imaging Multimode reader (BioTek Instruments, Agilent Technologies, Santa Clara, CA, USA) at 500 nm.

#### 2.6.3. LDH Release Assay for Cytotoxicity

To measure LDH release, the CyQUANT LDH Cytotoxicity Assay kit (Fluorescence; Thermo Scientific; Cat. No. C20300) was used. According to the manufacturer’s protocol, 50 μL of culture medium was removed from cell culture and placed into a well of a 96-well plate. 50 μL of reaction mixture was added to each media sample and allowed to incubate for 30 min at 37° C protected from light. After 30 min, 50 μL of stop reaction solution was added to each well. The absorbances at 490 nm and 680 nm were measured by the microplate reader (BioRad Laboratories, Hercules, CA, USA). The 680 nm absorbance value was subtracted from the 490 nm absorbance. The cytotoxicity was calculated using the formula: % cytotoxicity = [(EV-treated LDH activity—spontaneous LDH activity)/(maximum LDH activity—spontaneous LDH activity)] * × 100%.

#### 2.6.4. ROS Assay for Oxidative Stress

The cells were washed with PBS and treated with 25 μM carboxy-H_2_DCFDA (Invitrogen, Thermo Scientific; Cat. No. C400). After 30 min incubation at 37 °C protected from light, the cells were washed using PBS three times and resuspended in PBS. Relative fluorescence was measured by a Cytation 5 Cell Imaging Multimode reader (BioTek Instruments) at excitation and emission wavelengths of 495 nm and 529 nm, respectively.

### 2.7. Statistical Analysis

All experimental data were collected in at least triplicate (*n* = 3–4 independent biological replicates), and results are presented as the mean ± standard deviation (SD). Statistical analysis was performed using GraphPad Prism (Version 8.4.3, GraphPad Software, San Diego, CA). Pairwise comparisons between two experimental conditions were evaluated using an unpaired, two-tailed Student’s *t*-test. Differences across multiple experimental conditions within a single factor (e.g., qRT-PCR transcriptional analysis) were analyzed using a one-way analysis of variance (ANOVA) followed by Tukey’s post hoc test for multiple comparisons. Time-course EV concentration kinetics and size distributions involving two independent factors (treatment conditions and sampling time points) were evaluated using a two-way ANOVA with Tukey’s post hoc test to evaluate main treatment effects, interaction effects, and percent total variance partitioning. A *p*-value < 0.05 was considered statistically significant (* *p* < 0.05, ** *p* < 0.01, *** *p* < 0.001, **** *p* < 0.0001, ns = not significant).

## 3. Results

### 3.1. ES Enhances EV Secretion in ASCs

**Figure 2:**
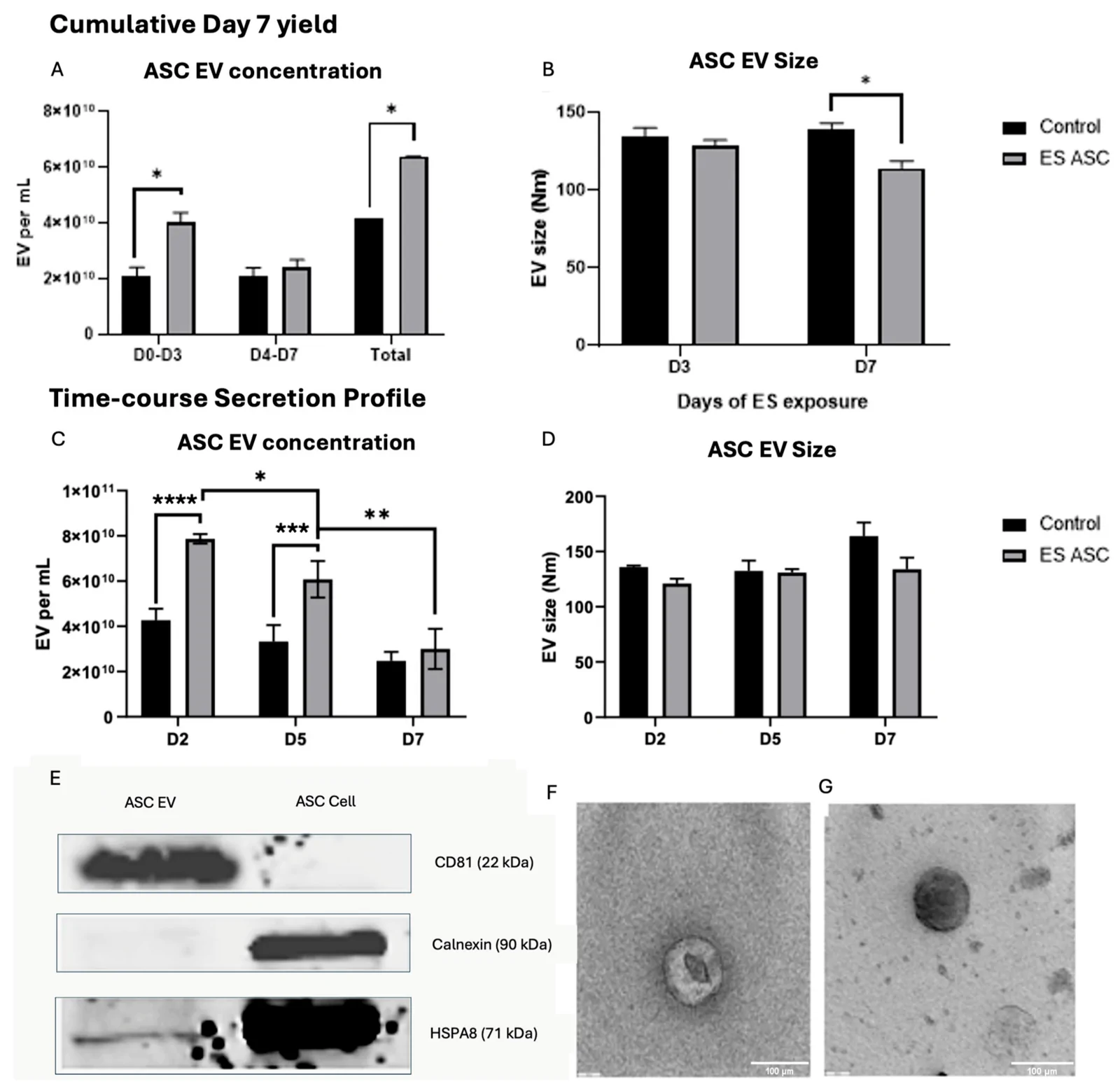
Characterization and quantification of EVs secreted by hASCs under ES. Comparative EV yield shows that ES treatment significantly increases the total concentration of EVs per mL of media compared to unstimulated hASCs: (**A**) cumulative day 7 yield; (**B**) EV size; (**C**) time-course EV secretion; (**D**) EV size using a second batch of hASCs; (**E**) protein expression analysis via Western blot, confirming the presence of EV markers. Morphology was assessed using transmission electron microscopy (TEM), revealing that ES-exposed to EVs (**F**) retain the characteristic “cup-shaped” morphology and membrane integrity observed in unstimulated hASC-derived EVs (**G**). N = 3–4 independent biological replicates. * indicates *p* < 0.05. ** indicates *p* < 0.01. *** indicates *p* < 0.001. **** indicates *p* < 0.0001.

To evaluate the efficacy of ES on EV secretion in primary stem cells, hASC-derived vesicles were analyzed for yield and size distribution via NTA across day a 7-day timeline (Figure 2). ES-treated hASCs demonstrated a significant increase in total EV secretion during the initial 72 h (days 0–3) compared to unstimulated controls (4 × 10^10^ vs. 2 × 10^10^ EVs/mL). Although secretion between days 4–7 was comparable, the cumulative 7-day EV yield was significantly higher in the ES group (6 × 10^10^ vs. 4 × 10^10^ EVs/mL; Figure 2A, Supplementary Figure S1). ES also modulated size distribution. While EV diameter was unaffected at day 3, ES-derived EVs were significantly smaller than controls by day 7 (100 nm vs. 140 nm; Figure 2B), suggesting temporal shifts in vesicle biogenesis dynamics.

To complement the cumulative endpoint analysis with independent data, a separate cohort of hASCs was evaluated in a time-course study measuring discrete, interval-specific secretome harvests (days 2, 5, and 7) rather than continuous 7-day accumulation. A time-course profile analyzed via two-way ANOVA confirmed that ES enhanced EV output most significantly at day 2 (*p* < 0.0001) and day 5 (*p* = 0.0002) compared to the unstimulated control, before returning to baseline levels by day 7 (*p* = 0.5820, ns) (Figure 2C). Variance partitioning revealed that while ES treatment accounted for 30.1% of overall secretion variation (*F*(1, 13) = 60.86, *p* < 0.001), time-dependent culture kinetics served as the primary determinant of EV yield in hASCs, accounting for 40.2% of total variation (*F*(2, 13) = 40.59, *p* < 0.001). A significant treatment *x* time interaction was also observed (8.1% of variance, *F*(2, 13) = 8.18, *p* = 0.0049), driven by a progressive decay in ES hyper-secretion kinetics across the 7-day culture period. Evaluation of EV size across these individual sampling intervals (Figure 2D and Supplementary Figure S2) demonstrated that while hydrodynamic diameter remained uniform through day 5, ES treatment preserved significantly smaller vesicle sizes at day 7 compared to control EVs (*p* = 0.0003). Western blot analysis confirmed the presence of positive exosomal markers CD81 and HSPA8/HSC70 alongside the absence of the negative marker calnexin (Figure 2E, Supplementary Figure S3). TEM images confirmed typical cup-shaped exosomal morphology in both ES and non-ES conditions (Figure 2F,G).

**Figure 3:**
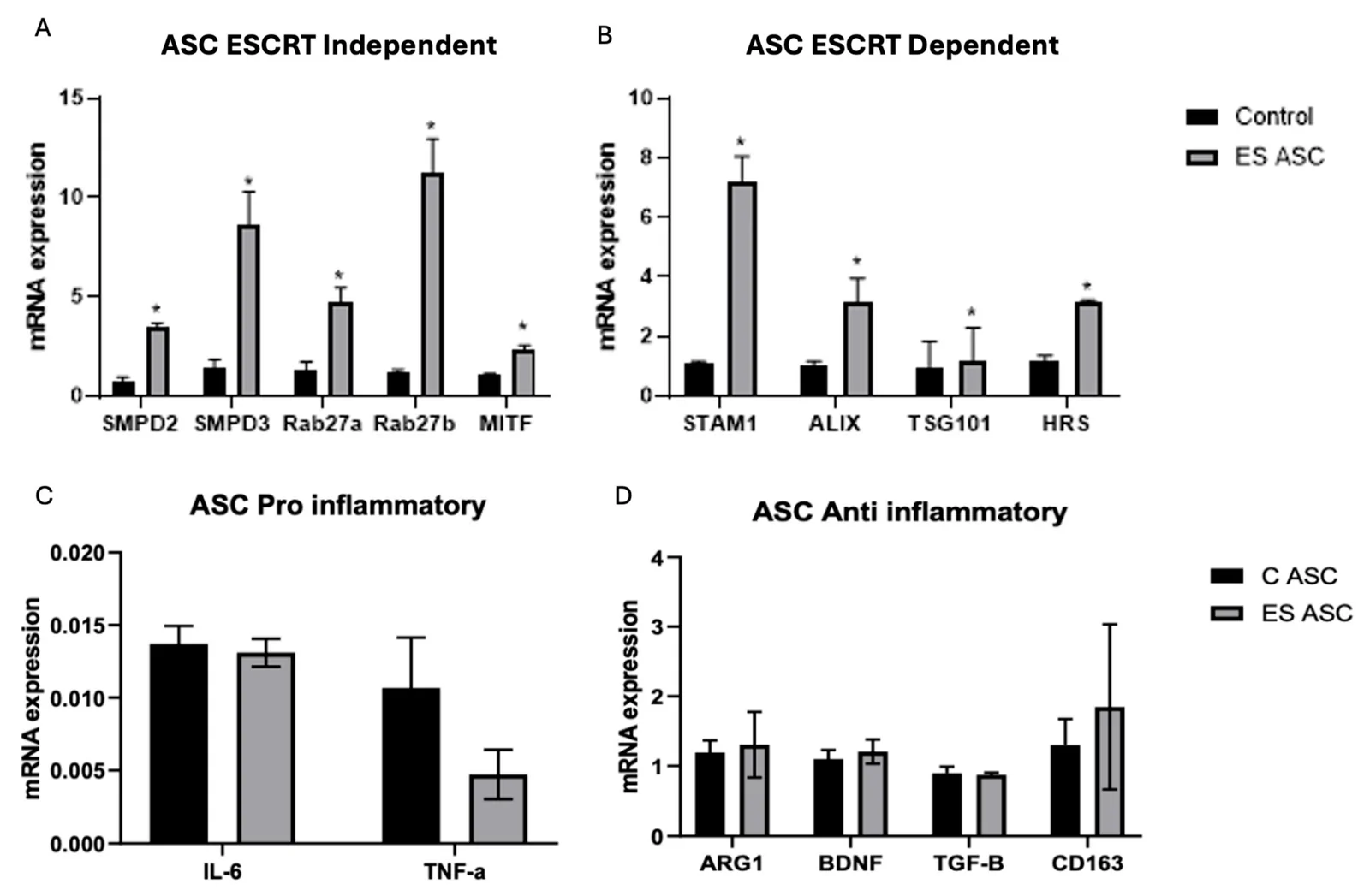
Transcriptional modulation of EV biogenesis and inflammatory markers in hASCs following ES. Biogenesis pathway analysis showing the relative mRNA expression of (**A**) ESCRT-independent genes and (**B**) ESCRT-dependent genes, indicating that molecular pathways were upregulated by electrical cues to drive EV production. (**C**) Pro-inflammatory gene expression. (**D**) Anti-inflammatory gene expression. N = 3. * indicates *p* < 0.05. ** indicates *p* < 0.01. *** indicates *p* < 0.001.

qRT-PCR analysis revealed that 100 mV ES triggered robust upregulation across both ESCRT-independent (*SMPD2*, *SMPD3*, *Rab27a*, *Rab27b*, *MITF*; 2–10-fold) and ESCRT-dependent pathways (*STAM1*, *ALIX*, *TSG101*, *HRS*; 3–7-fold; Figure 3A,B). Additionally, ES did not alter the cells’ immunomodulatory profile, showing no significant changes in pro-inflammatory (*IL-6*, *TNF*-*a*) or anti-inflammatory markers (*ARG1*, *TGB-B*, *CD163*; Figure 3C,D). Collectively, these findings indicate that ES increases EV yield alongside a concurrent transcriptional upregulation of key biogenesis-associated genes, while preserving the baseline hASC immunomodulatory phenotype.

### 3.2. Comparative EV Secretion Across Cell Types

**Figure 4:**
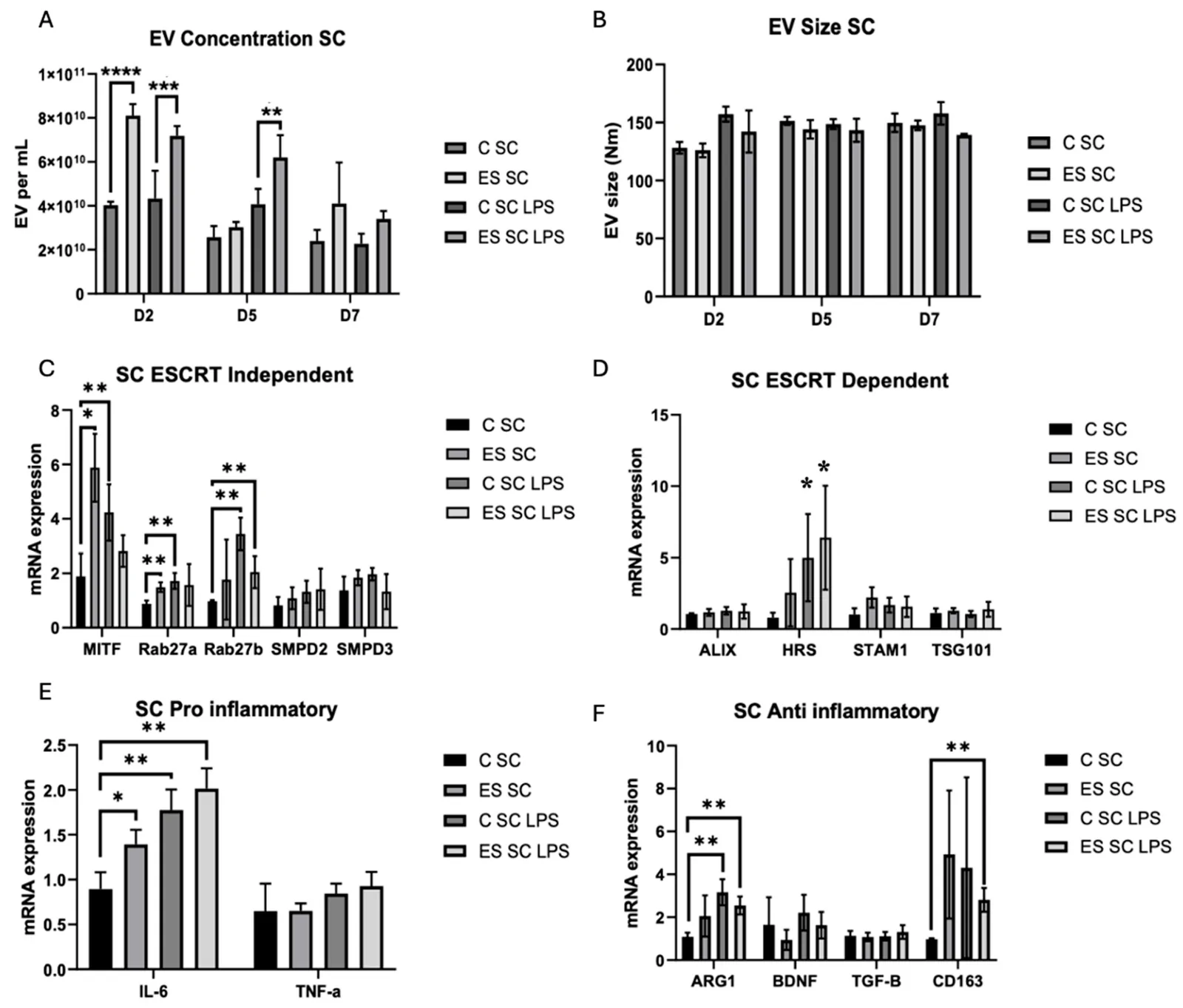
Physical and transcriptional characterization of hSC responses to ES and LPS-induced inflammation. Quantitative and physical EV characterization via NTA, showing shifts in (**A**) total EV concentration, and (**B**) a consistent size distribution regardless of LPS or ES treatment status. Biogenesis pathway modulation showing mRNA expression of (**C**) ESCRT-independent and (**D**) ESCRT-dependent genes across control, ES, LPS, and ES + LPS groups, illustrating the combined effect of electrical and inflammatory cues on EV production pathways. Immunomodulatory gene expression highlighting (**E**) the pro-inflammatory, and (**F**) anti-inflammatory transcriptional profiles; notably, ES helps mitigate the inflammatory markers spiked by LPS treatment. All datasets represent comparisons between control SC (unstimulated, untreated), electrically stimulated SCs (ES SC), LPS-treated SC (C SC LPS), and electrically stimulated LPS-treated SC (ES SC LPS). N = 3–4 indepednet biological replicates. * indicates *p* < 0.05. ** indicates *p* < 0.01. *** indicates *p* < 0.001. **** indicates *p* < 0.0001.

To evaluate ES effects on the native nerve environment, hSCs were analyzed under basal and LPS-induced inflammatory conditions. Time-course NTA evaluated using two-way ANOVA demonstrated that ES significantly promoted EV secretion across all conditions (Figure 4A). Variance partitioning revealed that, in contrast to stem cell secretome dynamics, treatment conditions was the primary determinant of EV yield in SCs, accounting for 42.6% of total variation (*F*(3, 24) = 48.32, *p* < 0.0001), outweighing time-dependent culture decay (27.5% of variation, *F*(2, 24) = 31.15, *p* < 0.001). A significant condition × time interaction was also identified (14.0% of variation, *F*(6, 24) = 7.94, *p* < 0.0001).

By day 2, ES induced a 2-fold increase in EV concentration compared to unstimulated controls in both basal (*p* < 0.001) and LPS conditions (*p* = 0.0004; 4 × 10^10^ to 8 × 10^10^ EVs/mL). While basal ES-stimulated cultures returned to control secretion rates by day 5 (*p* = 0.8840, ns), elevated yield persisted in LPS-treated ES groups at days 5 (*p* = 0.0080; 1.5-fold increase over unstimulated inflammatory controls) before returning to baseline levels across all groups by day 7 (*p* = 0.3120, ns). Overall EV diameter remained consistent (120–160 nm; Figure 4B), and hSCs maintained characteristic morphology (Supplementary Figure S4). TEM and Western blot confirmed exosomal morphology and marker expression (Supplementary Figure S5).

qRT-PCR profiling revealed distinct biogenesis responses across ESCRT pathways. In basal hSCs, ES significantly upregulated ESCRT-independent markers *MITF* (~3-fold) and *Rab27a* (~2-fold; Figure 4C). LPS exposure alone increased *MITF* (~2-fold) *Rab27a* (~2-fold), and *Rab27b* (~ 3.5-fold), indicating an inherent stress-induced vesicle response. Combining ES with LPS further elevated *Rab27b* (~2-fold over healthy control), whereas *SMPD2* and *SMPD3* were unaffected. Conversely, ESCRT-dependent markers (*ALIX*, *STAM1*, *TSG101*) showed no significant differences, except *HRS*, which was elevated (5–6-fold) in both LPS-treated groups regardless of ES (Figure 4D).

Immunomodulatory evaluation showed moderate *IL-6* upregulation (1.5–2.0-fold) across all treated groups relative to healthy controls, with no significant changes in *TNF-a* (Figure 4E). Among anti-inflammatory markers, *ARG1* increased 2–3-fold in both LPS groups, while *CD163* was significantly elevated in the ES + LPS group (Figure 4F). Thus, while LPS shifts hSCs toward an activated state, ES enhances high-yield EV biogenesis without intensifying pro-inflammatory signaling beyond LPS baseline levels.

### 3.3. hASC EV Treatment in an In Vitro Ischemic Stroke Model

**Figure 5:**
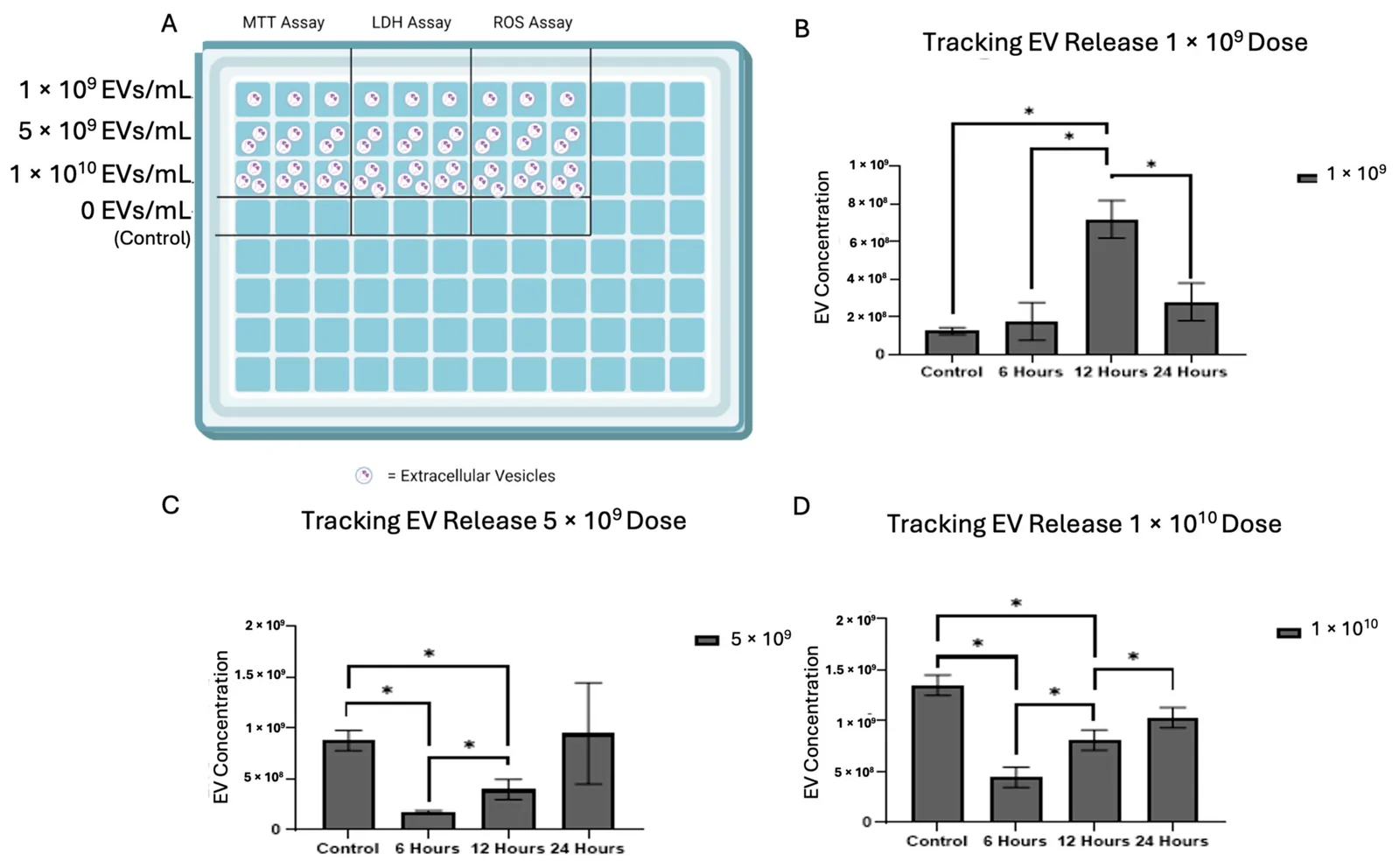
Encapsulation efficacy and sustained release kinetics of hASC-derived EVs in a 3D collagen hydrogel matrix. (**A**) Schematic illustration of the encapsulation process, demonstrating the loading of EVs into 3D collagen hydrogels at three distinct densities (1 × 10^9^, 5 × 10^9^, 1 × 10^10^ EVs/mL) to evaluate dose-dependent release profiles. Sustained EV release kinetics, showing a clear correlation between the initial loading density and the subsequent concentration of EVs released into the media: (**B**) 1 × 10^9^, (**C**) 5 × 10^9^, (**D**) 1 × 10^10^ EVs/mL. The 24 h pre-wash cycle ensures that the quantified particles represent sustained release from the hydrogel matrix. * indicates *p* < 0.05.

To evaluate the therapeutic efficacy of hASC-derived EVs, a 2 mg/mL collagen hydrogel delivery system composed of rat tail Type 1 collagen, 10× PBS, 1 N NaOH, and DI water was developed to mimic the biophysical elasticity and porosity required for localized EV delivery (Figure 5A). EVs were loaded into the hydrogel then solidified for 30–40 min at 37 °C. Initial NTA confirmed that isolated hASC EVs exhibited an average particle size of 255.5 ± 3.7 nm, a mode diameter of 193.8 ± 15.4 nm, and a stock concentration of 8.61 × 10^11^ EVs/mL.

Release kinetics were evaluated by measuring EV concentration in media supernatants at 6, 12, and 24 h following an initial wash (Supplementary Figure S6). For the 1 × 10^9^ EVs/mL dose group, EV release steadily increased over time, peaking at 12 h before slightly declining at 24 h (Figure 5B). At the 5 × 10^9^ EVs/mL dose, released EVs maintained a mean size of 216.8 nm and mode size of 184.2 nm, displaying a net increase in EV concentration from 6 to 24 h (Figure 5C). The 1 × 10^10^ EVs/mL dose exhibited the most consistent sustained-release profile, maintaining characteristic EV size parameters (mean: 227.2 nm; mode: 185.8 nm) with steadily increasing cumulative release through 24 h (Figure 5D).

### 3.4. Cell Viability, Cytotoxicity, and Oxidative Stress Assays

**Figure 6:**
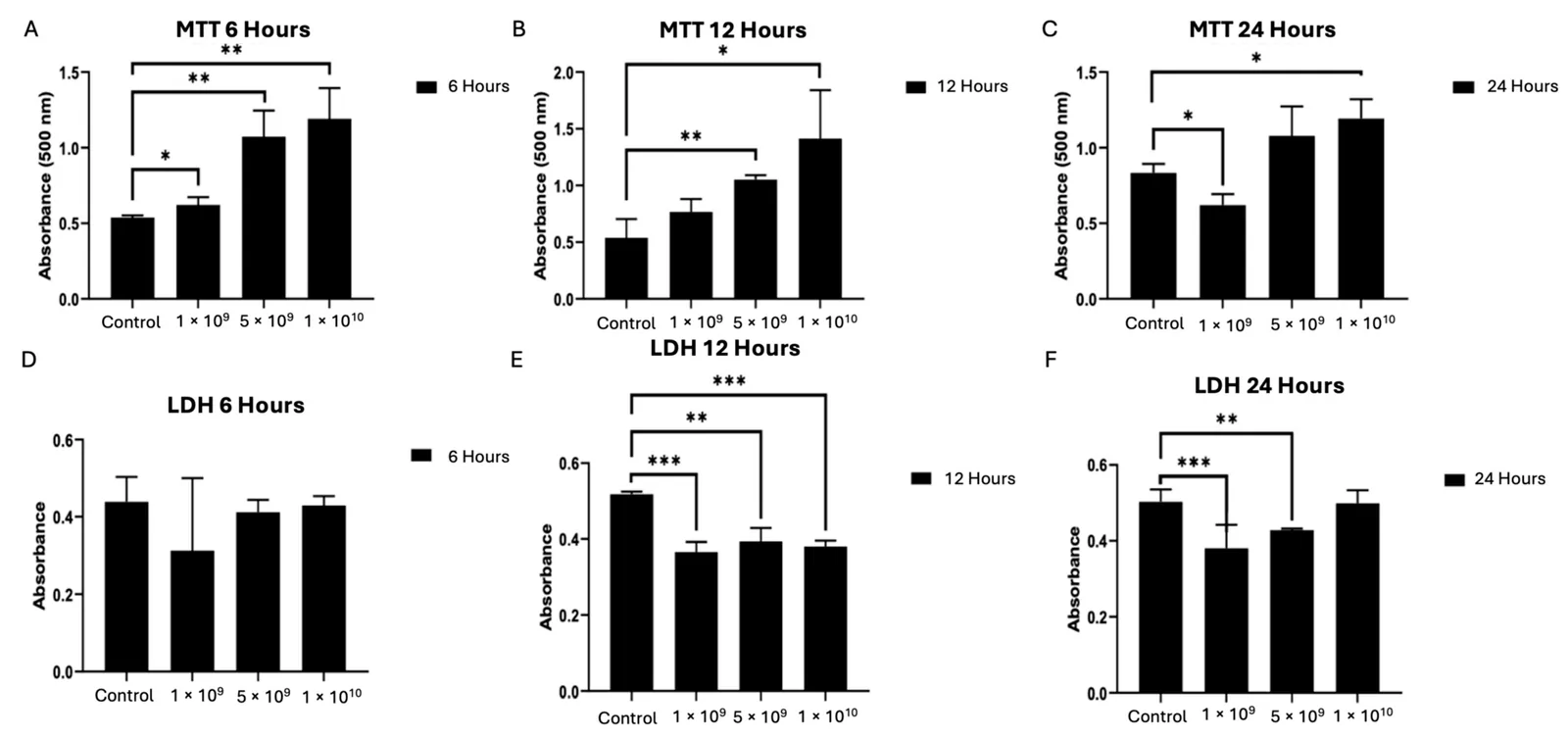
Therapeutic efficacy of hydrogel-delivered hASC-EVs in an in vitro ischemic stroke model. Metabolic recovery assessment via MTT assay, showing a significant, dose-dependent increase in cell viability at (**A**) 6 h, (**B**) 12 h, and (**C**) 24 h following hypoxic exposure when treated with EV-loaded hydrogels compared to untreated control. Cytotoxicity analysis measuring LDH release at (**D**) 6 h, (**E**) 12 h, and (**F**) 24 h, demonstrating that the sustained release of EVs significantly reduces cell membrane damage, specifically in the 12 and 24 h time points. N = 3. * indicates *p* < 0.05. ** indicates *p* < 0.01. *** indicates *p* < 0.001.

Cell viability and metabolic activity following hASC-derived EV treatment (1 × 10^9^, 5 × 10^9^, and 1 × 10^10^ EVs/mL) were evaluated after 6, 12, and 24 h of hypoxic exposure (Figure 6A–C). At 6 h, all EV doses significantly increased metabolic activity relative to control. At 12 h, the 5 × 10^9^ and 1 × 10^10^ EV/mL conditions maintained significantly elevated metabolic activity compared to control, whereas the 1 × 10^9^ EVs/mL dose showed a smaller increase. By 24 h, dose-dependent responses diverged: the 1 × 10^9^ EVs/mL group exhibited a significant reduction relative to control, whereas the 1 × 10^10^ EVs/mL group remained significantly elevated. No significant difference was observed between the 5 × 10^9^ EVs/mL and control at 24 h.

Cytotoxicity was quantified via LDH release (Figure 6D–F). At 6 h, LDH release was comparable between control and EV-treated groups. By 12 h, all EV treatment groups exhibited significantly reduced LDH release compared to control, with maximal reductions observed at 1 × 10^9^ and 1 × 10^10^ EV/mL. At 24 h, LDH release remained significantly reduced in the 1 × 10^9^ and 5 × 10^9^ EV/mL groups, while the 1 × 10^10^ EV/mL was not significantly different from the control. Oxidative stress analysis revealed that 1 × 10^9^ and 5 × 10^9^ EVs/mL hASC-EVs significantly reduced ROS levels at 12 h compared to control, with no significant difference detected at other time points (Supplementary Figure S7).

**Figure 7:**
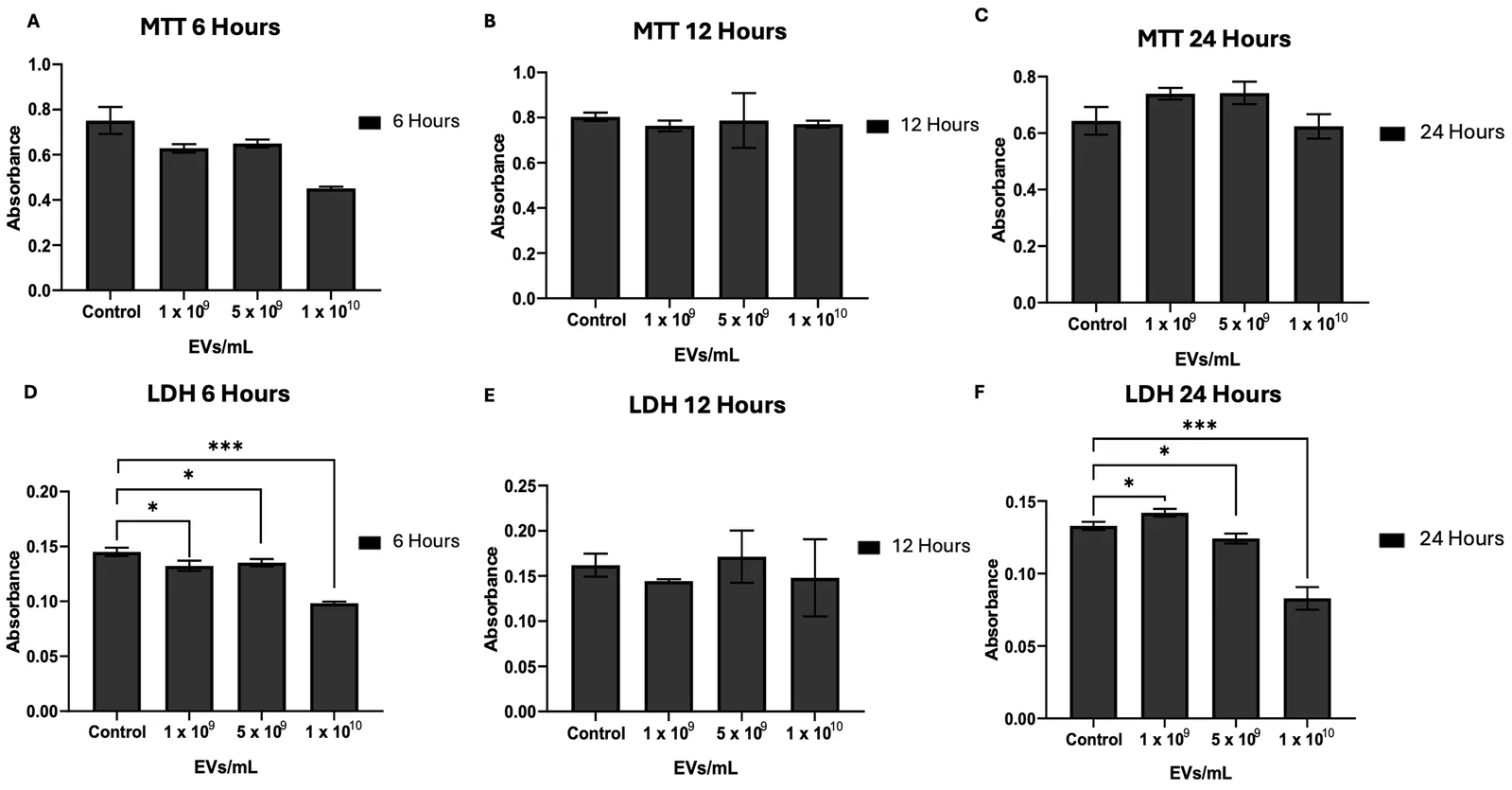
Therapeutic efficacy of hydrogel-delivered hSC-EVs in an in vitro ischemic stroke model. Metabolic recovery assessment via MTT assay at (**A**) 6 h, (**B**) 12 h, and (**C**) 24 h following hypoxic exposure when treated with EV-loaded hydrogels compared to untreated control. Cytotoxicity measured by LDH release at (**D**) 6 h, (**E**) 12 h, and (**F**) 24 h, demonstrating reduced LDH levels as EV concentration increased. N = 3. * indicates *p* < 0.05. ** indicates *p* < 0.01. *** indicates *p* < 0.001.

To assess source-dependent effects, cells were also treated with hSC-derived EVs at equivalent doses (Figure 7). Across 6, 12, and 24 h exposures, hSC-EVs produced no significant changes in overall metabolic activity via MTT assay (Figure 7A–C). In contrast, LDH assays demonstrated clear cytoprotective effects on membrane integrity (Figure 7D–F). At 6 h, all hSC-EV doses significantly reduced LDH release relative to control (Figure 7D). Although LDH levels were comparable across groups at 12 h (Figure 7E), all hSC-EV conditions demonstrated significantly lower LDH release than control by 24 h, with the greatest protection observed at the highest EV dose (Figure 7F). Together, these findings indicate that hSC-derived EVs primarily contribute to cytoprotection under hypoxic stress by preserving cell membrane integrity rather than modulating baseline metabolic rate.

## 4. Discussion

### 4.1. Effect of ES on EV Secretion

The ESCRT-independent pathway genes, including *SMPD2*, *SMPD3*, *Rab27a*, *Rab27b*, and *MITF,* facilitate cargo sorting and EV biogenesis; ESCRT-dependent pathway genes, including *STAM1*, *ALIX*, *TSG101*, and *HRS*, facilitate multivesicular body (MVB) fusion and exosome release [35]. In this study, ES was associated with elevated expression of key EV biogenesis markers in hASCs and hSCs. Assessment of pro- and anti-inflammatory gene expression suggested a stable immunomodulatory phenotype under the tested electrical parameters. Low cytokine expression indicates that hASCs maintained baseline homeostatic states under these specific stimulation conditions without triggering a strong acute pro-inflammatory response, supporting the prospective utility of ES as a low-cytotoxic method to enhance secretome activity. However, broader transcriptomic profiling and longer-term immune assays remain necessary to fully establish complete immunogenic stability.

Prolonged exposure to ES following three days of culture was hypothesized to contribute to the observed decline in EV production. Similar trends were reported by Hu et al. when investigating optimal ES parameters for dorsal root ganglion (DRG) nerve cells, where expanding stimulation from one to two hours reduced cellular viability [20]. This response was attributed to potential membrane hyper-depolarization and impaired ion channel kinetics required for cell maintenance [20]. Because EV biogenesis relies on dynamic membrane receptor signaling—including G protein-coupled receptors (GPCRs) and epidermal growth factor receptors (EGFRs) [36]—maintaining membrane integrity during external stimulation is critical. This electrochemical threshold offers a rationale for the observed plateau in EV concentration. Although initial ES promotes biogenesis, sustained exposure to a specific voltage may saturate secretory machinery or trigger a compensatory shift in metabolic activity. As a result, while cells may continue to generate EVs, net secretion stabilizes as cellular resources are redirected toward maintaining membrane integrity under sustained electrical stress.

Previous studies demonstrate that ES can be delivered via conductive hydrogels, electrospun nanofibers, and fluidic electrical fields, offering increased surface area for cellular attachment while mimicking native physiological microenvironments [37,38,39,40]. These 3D systems offer promising insights into scalability by indicating that electrical signals can penetrate complex matrices and influence dense cell populations. Nevertheless, a critical translation gap remains. Much of the existing ES literature focuses on in situ tissue regeneration and lineage-specific differentiation rather than ex vitro collection, isolation, functional characterization, and quantification of the secretome.

Furthermore, the transition to clinical manufacturing requires techniques that surpass the limitations of differential ultracentrifugation. Large-scale production of ES-enhanced EVs would benefit from high-throughput methods, such as Tangential Flow Filtration (TFF) and size-exclusion chromatography (SEC) [41]. TFF allows for the gentle concentration of large volumes of conditioned media while maintaining low-shear environment [42]. Subsequently, SEC facilitates a more precise separation of EVs from soluble proteins and small-molecule contaminants based on size [43]. In addition, EVs isolated using PEG-based methods may have low purity and be contaminated with cellular materials. Filtration-based methods can lead to high EV purity. Compared to established priming approaches—such as hypoxic preconditioning, 3D aggregate culture, or high-shear bioreactor expansion—low-voltage ES offers a non-invasive physical cue that enhances EV secretion while producing conditioned media with fewer cellular debris co-isolates, facilitating downstream purification for therapeutic applications [44].

### 4.2. Differences Between Cell Types and Response to ES

The distinct transcriptional and functional responses observed between hASCs and hSCs under ES highlight the importance of cell-type-specific mechanisms in bioelectric therapies. In this study, the inclusion of hSCs serves as a proof of concept to evaluate the broader applicability of ES as a therapeutic option. While hASCs represent a promising candidate for high-yield therapeutic EV production, characterizing hSC responses provides insight into how the native nerve neural microenvironment might respond to similar electrical stimuli. Previous studies indicate that ES can modulate intracellular signaling, cytoskeletal reorganization, and vesicle trafficking [45,46]. For instance, SCs exposed to low-level direct current exhibited an 11-fold increase in NGF release and significantly supported enhanced neurite outgrowth in co-culture systems [47]. Similarly, MSCs exposed to ES demonstrated altered neural differentiation and improved survival, often associated with activation of the PI3K/Akt and ERK signaling cascades [23].

Beyond direct neurotrophic support, SCs communicate locally via secreted vesicles that carry regulatory signaling molecules [48]. In rodent models, miRNA cargo within rSC-derived EVs has been shown to modulate axonal repair pathways. For example, López-Leal et al. demonstrated that rSC-derived EVs deliver miR-21 to injured neurons, promoting neurite elongation potentially via c-Jun and SOX2 pathway engagement alongside PI3K/Akt activation [49,50,51,52]. In these context-dependent mechanisms, c-Jun drives SC repair phenotypes, whereas SOX2 plays a key role in modulating immune responses, suppressing genes associated with myelination [49,50]. While these findings highlight the multifaceted role of hSCs in orchestrating peripheral nerve regeneration within rodent models, supporting the translation to human cellular models requires careful validation. Notably, while transcriptomic alterations under LPS + ES conditions suggest a transition toward a repair-supportive phenotype, transcript expression alone without protein-level validation (e.g., Western blot or immunostaining) warrants cautious interpretation. Future studies should focus on these phenotypic shifts at the protein level. By increasing EV yield in both progenitor and glial cells, this ES setup demonstrates that the hardware is a viable method for modulating the regenerative secretome in vitro. However, additional studies are necessary to evaluate whether these increases translate to therapeutic efficacy within the complex nerve microenvironment.

#### 4.2.1. EV Biogenesis Response

In hASCs, ES increased expression of both ESCRT-independent regulators (*Rab27a*, *Rab27b*, *SMPD2*, and *SMPD3)* and key ESCRT-dependent genes (*ALIX* and *STAM1*). Upregulation of *Rab27 a/b* and sphingomyelinase within electrically stimulated hASCs is consistent with enhanced Rab-dependent trafficking and ceramide-mediated vesicle inward budding, compared to the control and hSC groups [53,54,55]. This pattern suggests that ES may induce broad activation of EV formation machinery in hASCs, enhancing reliance on the ESCRT and ceramide-dependent routes for vesicle formation and release [54].

In contrast, the hSCs, particularly when exposed to LPS, exhibited localized transcript increases in genes linked to endosomal sorting and secretory/lysosomal programs (e.g., *HRS* and *MITF*) rather than broad, dual-pathway upregulation. This profile is consistent with reports identifying *MITF* as a key transcriptional regulator in repair SCs that drives lysosomal and secretory gene programs after axonal injury. Daboussi et al. demonstrated in rodent nerve injury models that *MITF* translocates to the nucleus to initiate downstream repair and lysosomal networks, whereas loss of *MITF* impaired SC transition to a repair phenotype and delayed functional recovery [56]. While our observations of elevated *MITF* expression in LPS- and ES-treated hSCs align with an activated transcriptional state, further protein-level verification is needed to confirm functional repair transition in human cells. Additionally, SC-derived EVs have potent immunoregulatory cargo within injured nerve models. Ren J. et al. demonstrated that rodent SC-derived EVs carrying MFG-E8 promoted macrophage transition toward an M2-like phenotype via SOCS3/STAT3 signaling, highlighting how glial secretome components may participate in resolving inflammatory microenvironments [57,58].

*HRS* expression was elevated in both baseline control and LPS-treated hSCs relative to hASCs. As a core subunit of the ESCRT-0 complex, *HRS* recognizes ubiquitinated cargo to initiate sorting into early endosomes prior to multivesicular body (MVB) maturation [35]. Rather than signaling a direct increase in total EV secretion, elevated *HRS* in hSCs may reflect an adaptive response to the injury-mimicking inflammatory environment. In this context, increased *HRS* expression could indicate that hSCs prioritize intracellular cargo routing and endosomal processing over high-volume exocytosis, which aligns with the known role of glia in immune modulation and debris management. This process is consistent with the hSC neuroprotective role following nerve injury. These expression patterns suggest that hSCs respond to inflammatory and bioelectrical cues by modulating specific endosomal trafficking networks rather than engaging a global secretome expansion.

Finally, relative stability in TSG101 expression across treatment conditions aligns with prior studies indicating that certain core ESCRT components operate at steady baseline levels, with their functional contribution varying by cell type and stimulus intensity [55]. Collectively, these transcriptional profiles suggest that ES in hASCs is associated with co-mobilization of ESCRT-dependent and ESCRT-independent biogenesis markers, whereas hSCs primarily alter specific endosomal and regulatory genes (*HRS*, *MITF*) that are characteristic of an injury-responsive, homeostatic glial phenotype.

#### 4.2.2. Inflammatory Response

Inflammatory gene expression further highlighted cell-type-specific responses to electrical and inflammatory stimuli. Among pro-inflammatory markers, *IL-6* transcript levels were significantly upregulated in control hSCs, ES-treated hSCs, control hSCs treated with LPS, and ES hSCs with LPS. This elevated *IL-6* expression is consistent with the expected natural role of hSCs in initiating an early immune response following injury. *IL-6* expression increased in a stepwise trend. Similarly, *TNF-α* expression was the highest in ES hSCs treated with LPS, following a stepwise trend similar to that of *IL-6*.

Anti-inflammatory genes revealed the most pronounced difference between the conditions. *ARG1*, *BDNF*, and *CD163* were elevated in hSCs across all ES and LPS conditions, with ES SC showing the highest *CD163* expression. This profile suggests that hSCs shift toward a pro-resolving or repair-supportive transcriptional phenotype under these stimuli, alongside altered neurotrophic gene expression. Notably, *BDNF* transcript expression peaked in control hSCs with LPS exposure, while TGFB remained relatively constant across all conditions. This selective upregulation indicates that ES and inflammatory cues modulates specific neuroprotective factors rather than inducing a uniform, global anti-inflammatory activity.

Consistent with the observed transcriptional responses, EV yield measurements indicated that both hASCs and hSCs exhibited enhanced EV production following ES exposure. EV concentration approximately doubled under ES conditions at early time points (day 2) in both cell types. This increased effect persisted through day 5. The LPS-stimulated hSCs produced the highest EV output overall. These findings indicate that while bioelectric cues provide an immediate biophysical trigger for EV exocytosis, combining ES with inflammatory priming synergistically expands the secretory capacity of hSCs, establishing early-to-mid culture intervals as the optimal harvest window to maximize EV yield.

Taken together, these findings highlight that ES enhances EV biogenesis in both hASCs and hSCs through distinct cellular and transcriptional responses. While hASCs primarily upregulate markers associated with both ESCRT-independent and ceramide-mediated trafficking pathways, hSCs—especially under inflammatory activation—leverage ESCRT-dependent and immunoregulatory mechanisms. The concurrent increase in anti-inflammatory and neurotrophic markers in hSCs further underscores their dual role as both immune modulators and facilitators of nerve repair. These differences in EV biogenesis and cytokine expression profiles have important implications for optimizing cell-specific stimulation strategies to enhance therapeutic EV production for neural regeneration.

### 4.3. Therapeutic Potential of the EVs

Ischemic stroke and the associated neuropathy remain a leading cause of mortality and long-term disability, resulting in irreversible damage to the central nervous system [59]. Current preventative and therapeutic strategies primarily rely on pharmacological agents, which are often associated with adverse side effects [59]. EVs derived from hMSCs have emerged as a promising alternative, offering the regenerative and immunomodulatory benefits of hMSCs without the risks inherent to cell-based therapy, such a vascular occlusion or uncontrolled proliferation [60].

Collagen hydrogels supported a sustained release of EVs over time, highlighting their potential use in extended-release applications. This release profile served as an important control, confirming that the gels functioned as a stable vehicle for gradual EV delivery. Importantly, the EVs released from the gels closely matched the size profile of those characterized during isolation. Furthermore, encapsulated EVs remained recoverable following extended incubation. Further optimization is needed to determine long-term release kinetics, degradation rates, and functional bioavailability in vivo.

Complementary in vitro assays were conducted to evaluate the functional impact of hydrogel-mediated EV delivery during cellular stress. The MTT assay measured metabolic activity, LDH release quantified cytotoxicity, and ROS production reflected intracellular oxidative stress. These metrics provide an in-depth perspective on how EVs influence cellular response to ischemic injury.

#### 4.3.1. Cell Metabolic Activity

The MTT assay was utilized as a direct indicator of cellular metabolic activity and mitochondrial respiration following OGD [61]. Across evaluated time points, hASC-EV-treated groups generally exhibited increased metabolic activity compared to non-treated OGD controls. In particular, cells receiving higher EV doses maintained significantly greater relative metabolic activity in a dose- and time-dependent manner, whereas lower-dose groups demonstrated reduced effects. These observations suggest that hASC-EV exposure supports cellular energy metabolism under simulated ischemic conditions.

The dose-dependent maintenance of metabolic activity observed at earlier time points suggests that hASC-EV treatment may support the capacity of cells to preserve mitochondrial function. Bioactive cargo within MSC secretomes, including miRNAs and mitochondria-associated proteins, have been reported to support oxidative phosphorylation and anti-apoptotic signaling [62,63]. These observations align with prior reports by Han et al. and Xiao et al., who similarly demonstrated that umbilical cord MSC-derived EVs significantly enhance MTT-measured metabolic viability and attenuate neuronal apoptosis under OGD conditions [64,65]. hASC-EV-treated cells retained higher relative MTT reduction compared to untreated controls, indicating that bioelectrically enhanced EVs may help protect cells against rapid metabolic collapse.

In contrast, treatment with hSC-derived EVs did not produce significant changes in MTT activity across the evaluated OGD time points. This suggests that SC-derived EVs may not primarily enhance global metabolic activity within the timeframe assessed. LDH release assays further evaluated whether hSC-EVs confer cytoprotection independent of direct metabolic evaluation.

#### 4.3.2. Cytotoxicity

Following cellular plasma membrane disruption during apoptosis or necrosis, intracellular LDH is released into extracellular space, serving as a marker of cytotoxicity [66]. In this study, intermediate EV concentrations (1 × 10^9^ and 5 × 10^9^ particles/mL) significantly attenuated LDH release at 24 h, suggesting that EV exposure helped preserve membrane integrity under hypoxic stress. In contrast, the highest EV dose (5 × 10^10^ EVs/mL) yielded LDH levels comparable to non-treated controls. Preclinical studies, such as that by Xie et al., similarly highlight that hMSC-derived EV administration reduces neuroinflammation and apoptosis in ischemic models, often operating through signaling networks including AMPK and JAK2/STAT3/NF-*k*B pathways [67].

hSC-derived EVs exhibited cytoprotection primarily through the preservation of cellular membrane integrity and attenuation of cytotoxic injury. Recent characterization of hSC-derived EVs indicates that these vesicles contain a diverse array of lipids, structural proteins, and regulatory miRNAs relevant to neural maintenance [68]. Work by Khan et al. demonstrated that hSC-EVs are enriched in membrane-associated lipids, such as phosphatidylcholine, which may contribute to plasma membrane repair, along with proteins and miRNAs linked to PI3K-Akt survival signaling [69]. Furthermore, literature demonstrates that hSC-derived EVs transport functional non-coding RNAs (IncARAT) that modulate recipient cell stress responses and axonal regeneration pathways following nerve injury [70,71]. These observations reinforce the concept that hSC-EVs exert bioactivity through regulatory and membrane-protective cascades rather than direct stimulation of cellular metabolism.

Interestingly, the 1 × 10^10^ EVs/mL dose increased metabolic activity while maintaining LDH levels near control values at 24 h. This observation highlights that higher EV doses may enhance mitochondrial function and metabolic activity without providing additional protection against membrane damage. Under ischemic stress, high EV exposure may stimulate mitochondrial metabolic activity in surviving cells without fully preventing plasma membrane compromise and downstream LDH release. Overall, these results underscore that elevated metabolic activity does not inherently reflect reduced cytotoxicity, reinforcing the importance of evaluating multiple functional endpoints when assessing EV-mediated cytoprotection.

#### 4.3.3. Oxidative Stress

Oxidative stress is a primary contributor to ischemic tissue injury, as excessive ROS accumulation damages cellular components and activates apoptotic and necrotic cascades [72]. In this study, the reduction in intracellular ROS levels observed at 12 h in the 1 × 10^9^ EVs/mL group suggests that moderate EV concentration provide early antioxidative protection under hypoxic stress. However, at 24 h, the 5 × 10^9^ EVs/mL group exhibited higher ROS signal, potentially reflecting a plateau in cellular benefit or a dose-dependent saturation threshold.

This attenuation of protective efficacy at higher particle densities or extended incubation aligns with established EV uptake kinetics in the literature. For example, Hansen et al. demonstrated that while fluorescently labeled EV uptake scales with dose and time, cellular internalization reaches a plateau after approximately 18 h due to endocytic saturation [73]. Similarly, Jurgielewicz et al. observed that EV uptake scales with dose up to approximately 6000 EVs/cell before declining at 24 h—an effect attributed to intracellular lysosomal processing, metabolic degradation, or recycling of internalized vesicles over extended timeframes [74]. Together, these studies suggest that while moderate EV doses can alleviate acute oxidative stress, excessive EVs or prolonged exposures trigger saturation or feedback mechanisms that limit protective capacity over time.

Collectively, these findings demonstrate that pre-treating cells with EVs prior to ischemia can enhance metabolic maintenance (MTT), attenuate plasma membrane disruption (LDH), and buffer acute oxidative stress (ROS). While the extent of protection is governed by dose-dependent windows and endocytic uptake constraints, these results support hydrogel-mediated EV delivery as a promising pre-conditioning strategy to mitigate acute hypoxic injury.

### 4.4. Limitations and Future Directions

While these findings provide preliminary evidence that ES can influence EV secretion profiles and parental cell biogenesis and inflammation, several limitations must be considered when interpreting these conclusions.

First, the electrical parameters evaluated in this study represent a discrete set of conditions rather than a fully optimized regime for sustained EV production. Previous research demonstrates that variations in voltage, frequency, and stimulation duration produce distinct biological responses and cargo profiles [22]. For instance, Wang et al. showed that electric field intensity regulates EV release kinetics and cargo loading in a frequency-dependent manner [22]. Because systematic parameter optimization was beyond the scope of this work, future studies should systematically evaluate voltage ramping profiles, pulse durations, and stimulation frequencies to determine if a higher yield or altered cargo potency can be achieved without inducing cellular stress or altering vesicle integrity.

Second, the observed plateau in EV concentration between days 5 and 7 suggests potential desensitization to fixed electrical parameters or culture-induced stress over time. Additionally, because media exchange occurred at fixed harvest intervals (days 2 and 5), the accumulation of metabolic waste products or altered media pH during 48 h windows may have influenced basal cellular secretion rates. Future time-course experiments incorporating routine metabolite tracking and variable recovery intervals will be necessary to clarify whether adaptive cellular responses or culture microenvironment shifts constrain continuous EV yield over extended culture periods.

Third, while in vitro readouts indicated changes in metabolic activity, membrane permeability, and reactive oxygen species levels, these assays measure general cellular survival metrics rather than definitive functional neural repair. Furthermore, because specific intra-vesicular cargo components were not individually knocked down or blocked, observed cytoprotective effects cannot be definitively attributed to a single molecular pathway or specific EV subpopulation. Incorporating complementary functional models—such as neurite outgrowth assays, electrophysiological recordings, or live calcium imaging—would provide deeper insight into whether these viability improvements translate to restored cellular function [75,76]. For instance, Ma et al. demonstrated that MSC-EVs promote neurite elongation in OGD-injured neurons [76], while Turovsky et al. utilized calcium imaging to confirm attenuation of intracellular Ca^2+^ overload, thereby promoted neurite growth through activation of the PI3K/Akt pathway [75]. Integrating similar functional endpoints will be essential to establish whether the observed in vitro cytoprotection reflects functional biological recovery.

Finally, while gene expression changes were observed in parent cells following ES, comprehensive proteomic and miRNA profiling of the secreted EVs was not performed across all stimulated conditions. Crucially, an increase in total EV yield does not inherently guarantee the production of a safer EV or one enriched with therapeutic factors. As a form of physical donor-cell manipulation, bioelectric stimulation can alter membrane physiology, stress-response pathways, and cargo sorting mechanisms—potentially resulting in unintended loading of stress-related proteins (such as heat shock proteins), inflammatory mediators, or altered nucleic acid profiles [77,78]. Consequently, direct claims regarding specific cargo enrichment or selective sorting of neuroprotective factors remain speculative. High-throughput sequencing and mass spectrometry of EV cargo—coupled with validation in relevant in vivo ischemic models—will ultimately be required to determine whether bioelectric stimulation selectively enriches functional subpopulations of vesicles with neuroprotective or anti-inflammatory factors. Beyond scaling particle quantity, successfully transitioning this bioelectric platform toward clinical translation will require closed-loop parameter monitoring, cGMP-compliant manufacturing, and standardized potency assays to guarantee batch-to-batch functional consistency across production runs [79].

## 5. Conclusions

This study demonstrated that low-frequency DC ES modulates EV biogenesis and inflammatory gene expression in both hASCs and hSCs. ES increased EV yield during the early stimulation period, with hASCs showing higher expression of ESCRT-dependent and ESCRT-independent genes involved in vesicle formation, while SCs—particularly under LPS activation—displayed upregulation of genes associated with anti-inflammatory signaling. These findings suggest that ES elicits distinct physiological responses within tissue repair and regeneration. Together, these results support ES as a promising, scalable, and non-invasive approach to enhance EV production, offering a practical solution to one of the major limitations of EV-based therapeutics: low yield. Moreover, the data reinforces the therapeutic potential of cell-free strategies that leverage hMSC- and hSC-derived EVs for treating peripheral neuropathy and related neuroinflammatory conditions. Continued optimization of stimulation parameters and EV characterization will be essential for translating these findings toward clinical applications.

## Data Availability

The data presented in this study are available on request from the corresponding author.

## Supplementary Materials

The following supporting information can be downloaded at: https://www.mdpi.com/article/10.3390/bioengineering13091025/s1. Supporting information: Number of pages: 12. Supplementary Figure S1. Size distribution of the extracellular vesicles measured by nanoparticle tracking analysis (Cumulative Day 7 yield). Supplementary Figure S2. Size distribution of the extracellular vesicles measured by nanoparticle tracking analysis (Time-Course Secretion Profile). Supplementary Figure S3. Additional western blot data for ASC EV marker expression. Supplementary Figure S4. Representative images of cells during the culture duration of the experiment on Days 2, 5, and 7. Supplementary Figure S5. hSC EV TEM and western blot data. Supplementary Figure S6. Representative size distribution of EVs used in functional assays to evaluate EV delivery across hydrogel dose conditions. Supplementary Figure S7. Intracellular ROS levels measured following exposure to EVs under hypoxic conditions. Supplementary Table S1. A list of antibodies. Supplementary Table S2. Primer information for qRT-PCR analysis.
