# Supplementary material for: Electrical Stimulation of Human Adipose Tissue-Derived Mesenchymal Stem Cells and Schwann Cells for Regulating Extracellular Vesicle Biogenesis and Inflammation
Source: Bioengineering (Basel). 2026 Sep 2;13(9):1025. doi: 10.3390/bioengineering13091025 (PMC13603633; doi:10.3390/bioengineering13091025)

Supplementary Materials

Electrical Stimulation of Human Adipose Tissue-Derived Mesenchymal Stem Cells and Schwann Cells for Regulating Extracellular Vesicle Biogenesis and Inflammation

Danyale Berry [1,2], Aakash Nathani [3], Fernando Carrillo [4], Abby Scott [4], Justice Ene [4], Colin Esmonde [4], Mandip Singh [3], Yan Li [4,*] and Changchun Zeng [1,2,*]

[1] Department of Industrial and Manufacturing Engineering, FAMU-FSU College of Engineering, Florida Agricultural and Mechanical University, Tallahassee, FL 32310, USA; danyale1.berry@famu.edu

[2] High Performance Materials Institute, FAMU-FSU College of Engineering, Florida State University, Tallahassee, FL 32310, USA

[3] College of Pharmacy and Pharmaceutical Sciences, Florida Agricultural and Mechanical University, Tallahassee, FL 32307, USA; aakash1.nathani@famu.edu (A.N.); mandip.sachdeva@famu.edu (M.S.)

[4] Department of Chemical and Biomedical Engineering, FAMU-FSU College of Engineering, Florida State University, 2525 Pottsdamer St., Tallahassee, FL 32310, USA; fc20a@fsu.edu (F.C.); ars20@fsu.edu (A.S.); je17d@fsu.edu (J.E.); cme18b@fsu.edu (C.E.)

* Correspondence: yli4@fsu.edu or yli@eng.famu.fsu.edu (Y.L.); zeng@eng.famu.fsu.edu (C.Z.); Tel: +1-850-410-6320 (Y.L.); Fax: +1-850-410-6150 (Y.L.)

Supporting information:

Number of pages: 12

Table of contents

Supplementary Figure S1. Size distribution of the extracellular vesicles measured by nanoparticle tracking analysis (Cumulative Day 7 yield).

Supplementary Figure S2: Size distribution of the extracellular vesicles measured by nanoparticle tracking analysis (Time-Course Secretion Profile).

Supplementary Figure S3: Additional western blot data for ASC EV marker expression.

Supplementary Figure S4: Representative images of cells during the culture duration of the experiment on Days 2, 5, and 7.

Supplementary Figure S5: hSC EV TEM and western blot data.

Supplementary Figure S6: Representative size distribution of EVs used in functional assays to evaluate EV delivery across hydrogel dose conditions.

Supplementary Figure S7: Intracellular ROS levels measured following exposure to EVs under hypoxic conditions.

Supplementary Table S1. A list of antibodies.

Supplementary Table S2. Primer information for qRT-PCR analysis.

Supplementary Table S1. A list of antibodies.

**Markers**	**Primary Antibody**	**Origin/ Isotype**	**Supplier/ Cat#**	**Dilution**
Exosomal markers	HSC70	Rabbit IgG	Rockland (VWR RL 100-401-F49)	Western Blot: 1:1000
	CD81	Rabbit IgG	Cell Signaling Technologies, #56039	Western Blot: 1:1000
	HRS	Rabbit IgG	Cell Signaling Technology, 15087	Western Blot: 1:1000
	TSG101	Mouse IgG_1	GeneTex, GTX70255	Western blot: 1:1000
	Calnexin (Negative exosome marker)	Rabbit IgG	GeneTex, GTX101676	Western Blot: 1:1000
Secondary	IRDye® 800CW	Goat anti-Rabbit IgG (H+L)	LI-COR/926-32211	Western blot: 1:5,000
	Alexa Fluor™ 488	Goat anti-Rabbit IgG (H + L)	Invitrogen, A-11008	Immunocytochemistry and Flow cytometry: 1:400

Supplementary Table S2. Primer information for qRT-PCR analysis.

Gene name	Primer sequence (5'-3')
h-ACTB	GTACTCCGTGTGGATCGGCG
	AAGCATTTGCGGTGGACGATGG
h-PDK1	AAACAGGGGAGCTTTGTCTGG
	CTGCCCATTCACATCCCTCTA
h-HK2	TGGTGTAGCTCCTCTGCTGCT
	TGTGGGCACCCTTTAGTGAAC
h-PKM2	AAAAATGGATGCCCAGAGGAC
	GAGTCGGCTTCAATGGAACAA
h-LDHA	CCTTGAGCCAGGTGGATGTTT
	CACTGGATCCCAGGATGTGAC
h-G6PD	CTACCCGAGCCCAGCTACATT
	TTCTGTTGGGCTGGAGTGAGT
h-6PGD	CCATGCCCTGTTTTACCACTG
	AGGTGTGAGCCCCGAAGTAAT
h-TALDO1	CTGTCATCAACCTGGGAAGGA
	GGGCGAAGGAGAAGAGTAACG
h-TKTL1	ACCTTGGGATTCTGTGTGCTG
	CCTAACAAGCTTTCGCTGCTG
h-SMPD2	GCCTGGGAGACTTTCTGAACC
	AAGTGGTGTGCAGCTGGGTAG
h-SMPD3	TTAAGAGACTCCAGGGCTGCTC
	CGGGGATTGTCAAAAACAGTC
h-SRSF5(HRS)	CTTCTCGGATCGAGGCTTCTT
	TCGAATCAACTGCGCTCATTA
h-STAM1	CACTGGATTTTTGGGTTGCTC
	GTGGAAAACATTTTTCGCATGA
h-TSG101	CACCTGGTGGTCCATATCCTG
	GATGGTGTCCTCGCTGATTGT
h-PDCD61P (ALIX)	TAAGTGCATCTGAGGGCCAAA
	GGGGCCTCCTTTCCTAGTTTC
h-RAB27A	GCATGTTTCAGTTTTCAAGAACCA
	AAAGGTGGCTTTTGTGTGTGC
h-RAB27B	TCCATGAAGCTGCTTGTCTCA
	GTTGGGTCTCCACCCAGAAAT
h-IL-6	ACCCCCAATAAATATAGGACTGGA
	GGAGTTCATAGCTGGGCTCCT
h-BDNF	AATTTTGCTCCCCAGTGAAGG
	GGCTCCCAACTTGACTTCTCC
h-CD163	CCAGTCCCAAACACTGTCCT
	ATGCCAGTGAGCTTCCCGTTCAGC
h-Arg (Arginase)-1	GGCAAGGTGATGGAAGAAACA

	GTGTGCCAGTAGCTGGTGTGA
h-TNF-a	CGTCGTAGCAAACCACCAAG
	TTGAAGAGAACCTGGGAGTAGACA
h-TGF-β	GAAGAACTGCTGCGTGCGGC
	CTGCGTGTCCAGGCTCCAAATG

Supplementary Figure S1. Size distribution of the extracellular vesicles measured by nanoparticle tracking analysis. (A) EVs derived from Experiment 1, Control ASC. (B) EVs derived from Experiment 1, electrically stimulated ASCs.

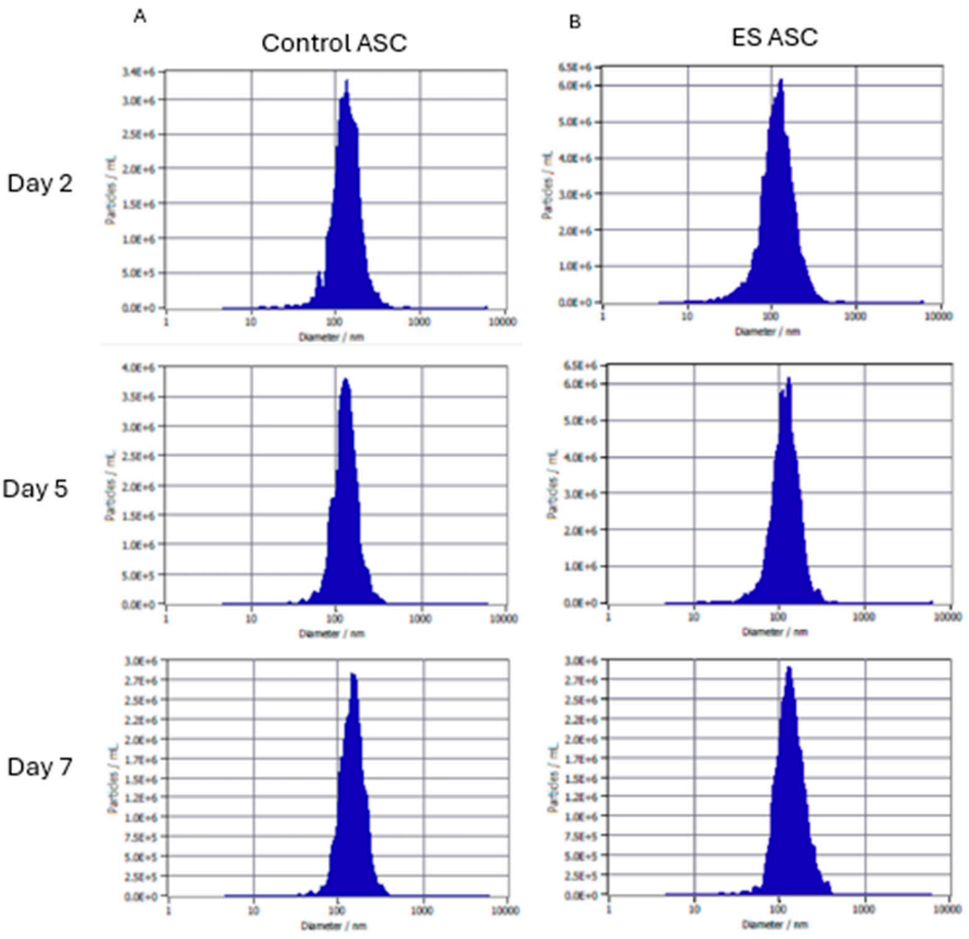

Supplementary Figure S2: Size distribution of the extracellular vesicles (EVs) measured by nanoparticle tracking analysis. EVs were derived from Experiment 2 and include: control Schwann cells without electrical stimulation (ES), Schwann cells exposed to ES, Schwann cells exposed to ES, Schwann cells treated with LPS, and Schwann cells treated with LPS and exposed to ES. Panels (A-D) correspond to these conditions, respectively.

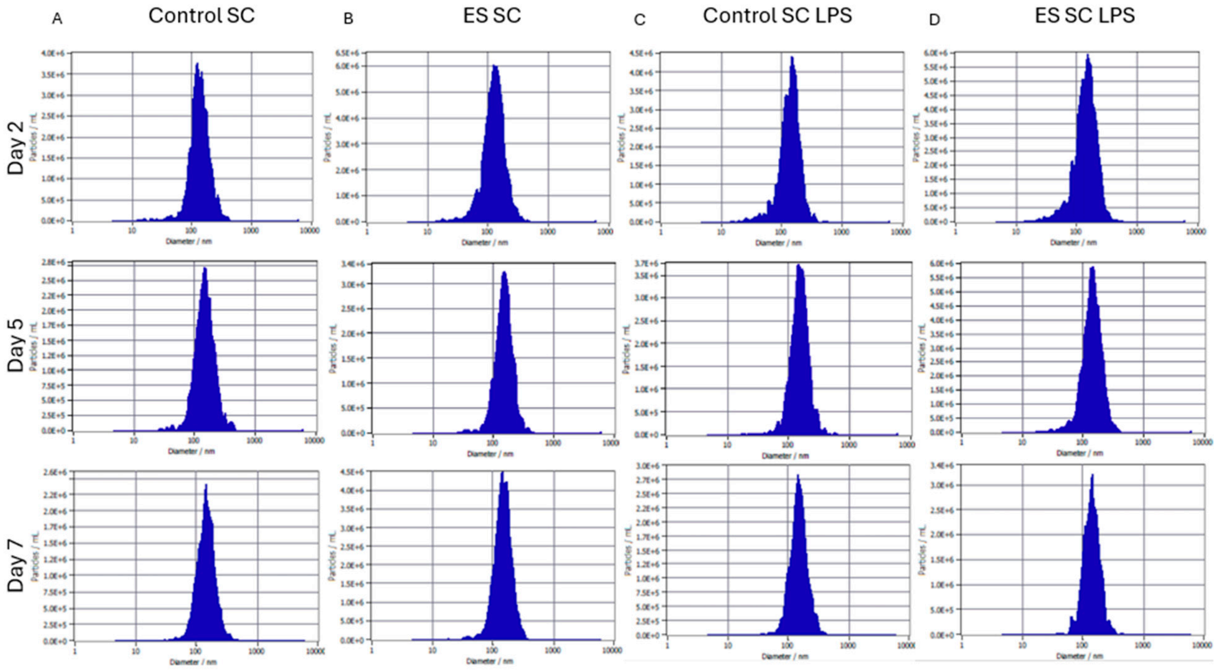

Supplementary Figure S3: Additional Western blot data for ASC EV marker expression.

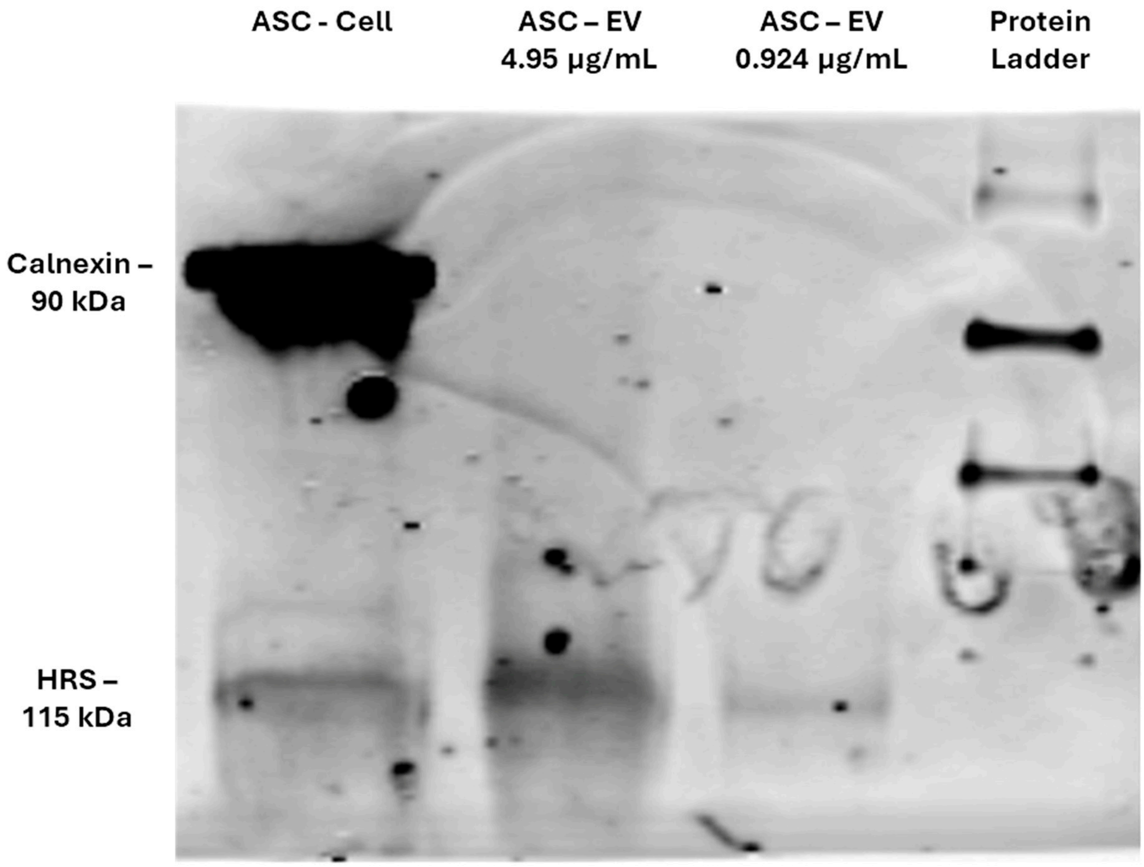

Supplementary Figure S4: Representative images of cells during the culture duration of the experiment on Days 2, 5, and 7. Images were captured at 10x and 20x magnification to illustrate cell morphology within 6-well plates**.** Panels show: (A) Control ASCs, (B) ES ASCs, (C) control SCs, (D) ES SCs, (E) control SC treated with LPS, and (F) ES SCs treated with LPS. ES: electrical stimulation. Scale bar: 100 µm for 10x and 50 µm for 20x.

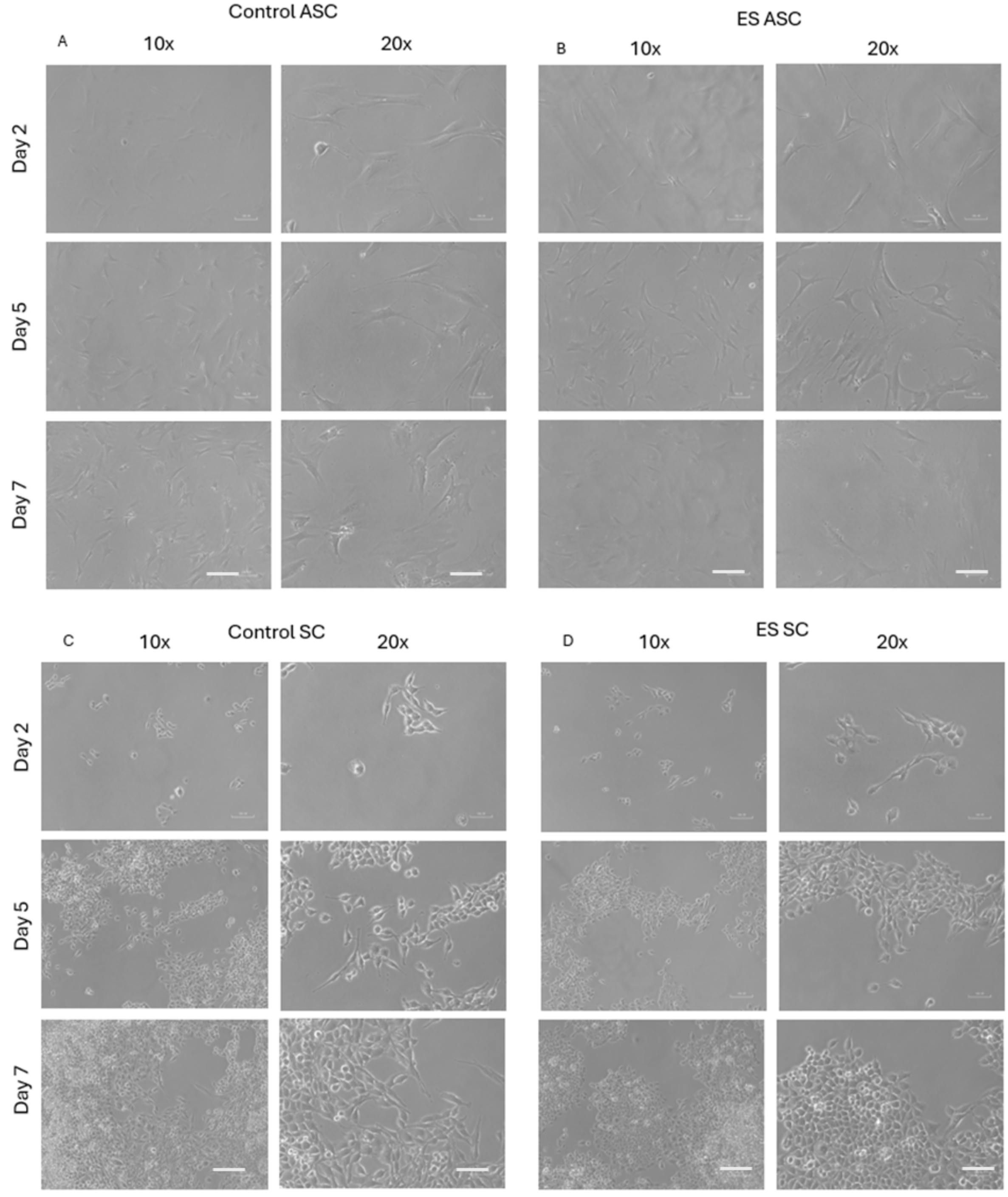

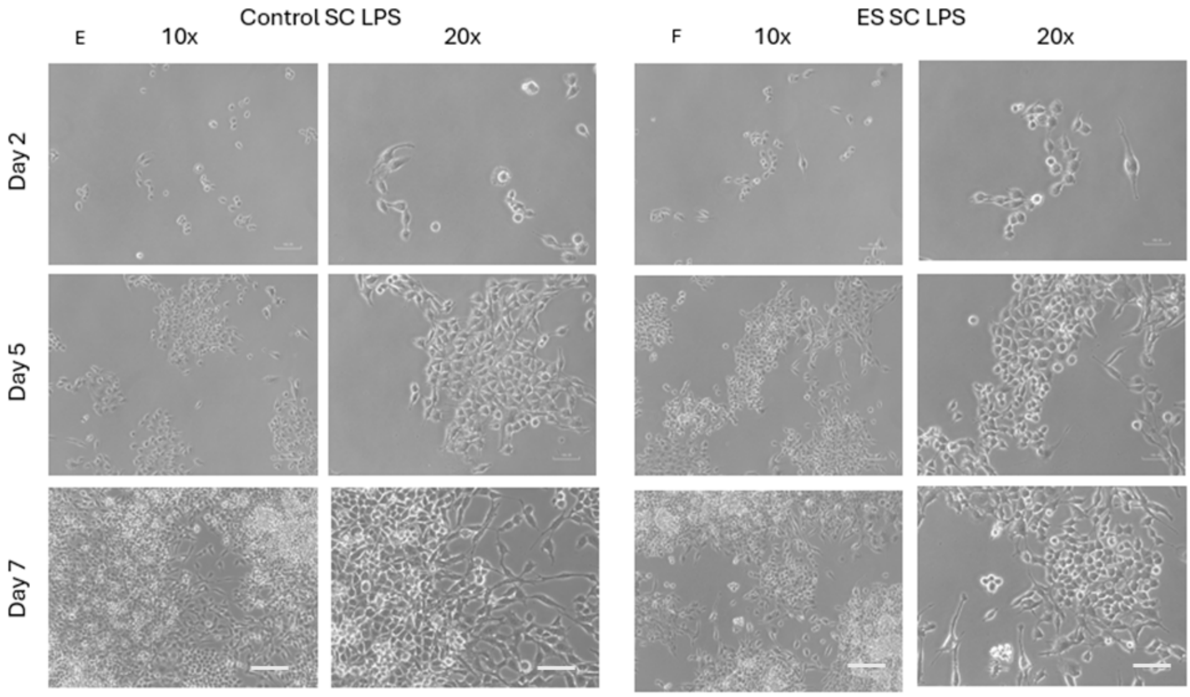

Control SC LPS
E
10x
20x
ES SC LPS
F
10x
20x
Day 2
Day 5
Day 7

Supplementary Figure S5. Characterization of human Schwann cell (hSC)-derived EVs. ((A) Western blot characterization of hSC-EVs. EV-associated markers TSG101 and HSC70 were detected in SC-EV samples. (B) Transmission electron microscopy (TEM) image of isolated EVs demonstrating the characteristic EV morphology, including spherical vesicles with cup-like structures. Scale bar of 60 nm.

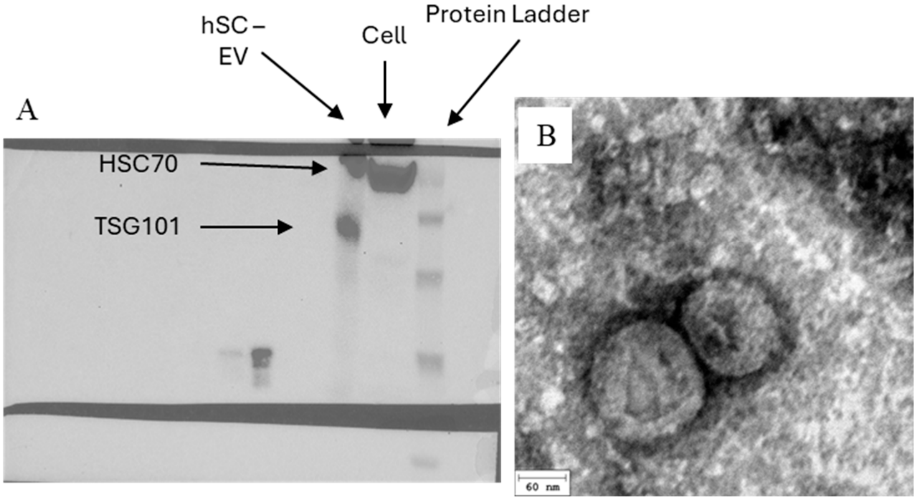

Supplementary Figure S6: Representative size distribution of EVs used in functional assays to evaluate EV delivery across hydrogel dose conditions. Panel (A) shows EVs collected during the 24-hour wash cycle prior to the experiment, used to quantify EV loss and confirm retention after hydrogel stabilization. Panels (B-D) show EVs released from the hydrogel at 6, 12, and 24 hours, respectively.

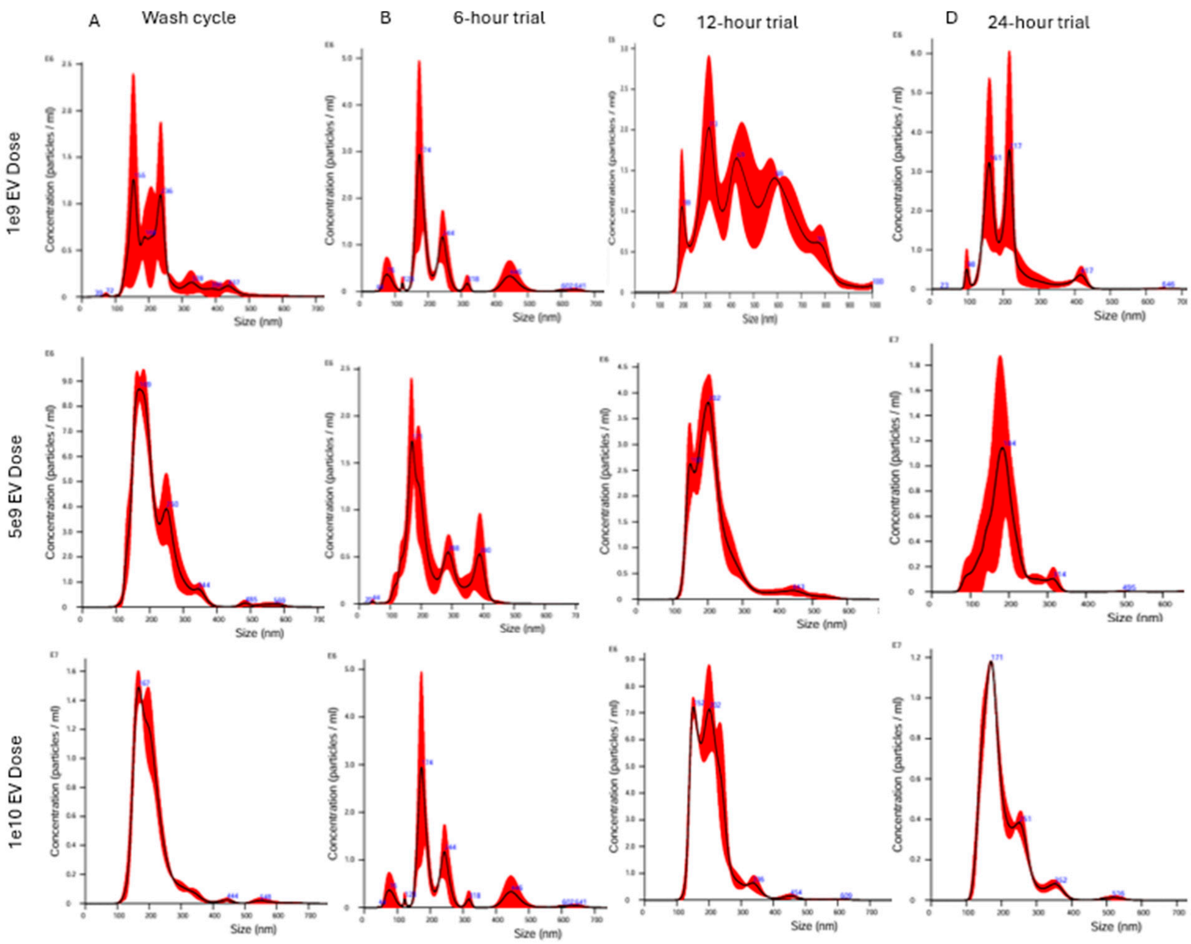

Supplementary Figure S7: Intracellular ROS levels measured following exposure to EVs under hypoxic conditions. ROS was quantified after 6, 12, and 24 hours of hypoxia for control, 1×10^9, 5×10^9, and 1×10^{10} EVs/mL conditions. N=3. * indicates $p<0.05$. ** indicates $p<0.01$. *** indicates $p<0.001$.

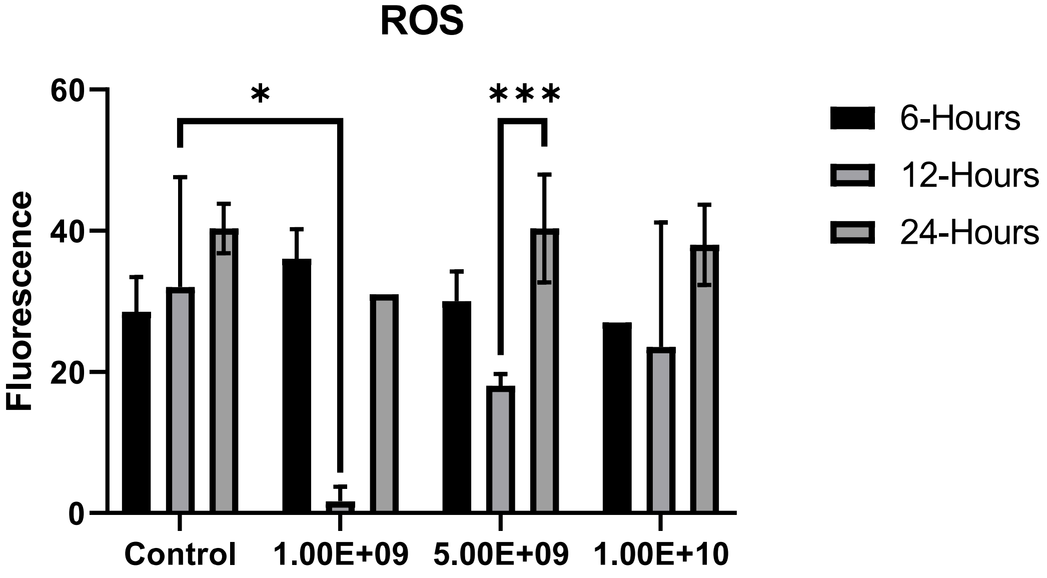

Supplement: Supplementary file 1 [file bioengineering-13-01025-s001.zip › bioengineering-4433838-supplementary.pdf]
